# A New Acoustic Portal into the Odontocete Ear and Vibrational Analysis of the Tympanoperiotic Complex

**DOI:** 10.1371/journal.pone.0011927

**Published:** 2010-08-04

**Authors:** Ted W. Cranford, Petr Krysl, Mats Amundin

**Affiliations:** 1 Department of Biology, San Diego State University, San Diego, California, United States of America; 2 Department of Structural Engineering, University of California San Diego, La Jolla, California, United States of America; 3 Department of Research and Education, Kolmarden Wildlife Park, Kolmarden, Sweden; 4 Department of Physics, Chemistry and Biology, Linköpings University, Linköping, Sweden; Raymond M. Alf Museum of Paleontology, United States of America

## Abstract

Global concern over the possible deleterious effects of noise on marine organisms was catalyzed when toothed whales stranded and died in the presence of high intensity sound. The lack of knowledge about mechanisms of hearing in toothed whales prompted our group to study the anatomy and build a finite element model to simulate sound reception in odontocetes. The primary auditory pathway in toothed whales is an evolutionary novelty, compensating for the impedance mismatch experienced by whale ancestors as they moved from hearing in air to hearing in water. The mechanism by which high-frequency vibrations pass from the low density fats of the lower jaw into the dense bones of the auditory apparatus is a key to understanding odontocete hearing. Here we identify a new acoustic portal into the ear complex, the tympanoperiotic complex (TPC) and a plausible mechanism by which sound is transduced into the bony components. We reveal the intact anatomic geometry using CT scanning, and test functional preconceptions using finite element modeling and vibrational analysis. We show that the mandibular fat bodies bifurcate posteriorly, attaching to the TPC in two distinct locations. The smaller branch is an inconspicuous, previously undescribed channel, a cone-shaped fat body that fits into a thin-walled bony funnel just anterior to the sigmoid process of the TPC. The TPC also contains regions of thin translucent bone that define zones of differential flexibility, enabling the TPC to bend in response to sound pressure, thus providing a mechanism for vibrations to pass through the ossicular chain. The techniques used to discover the new acoustic portal in toothed whales, provide a means to decipher auditory filtering, beam formation, impedance matching, and transduction. These tools can also be used to address concerns about the potential deleterious effects of high-intensity sound in a broad spectrum of marine organisms, from whales to fish.

## Introduction

**“Objects were made to vibrate. There are resonances hidden inside every lump and shard of nature.”** Mathieu (1991) [1]

Hearing in dolphins was one of the first subjects addressed by early cetacean research teams [2]–[5] because they suspected that dolphins, like bats, used echolocation [6]. In the following five decades, several review papers on hearing and ear anatomy in toothed whales (odontocetes) have been published [7]–[14].

**Figure 1:**
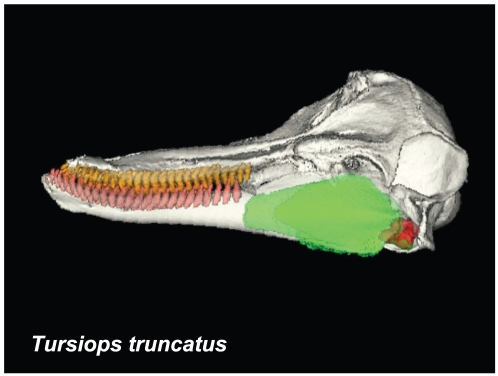
Left lateral view of the Atlantic Bottlenose Dolphin (*Tursiops truncatus*) from CT reconstructions. Skull = ivory, maxillary teeth = orange, mandibles = ivory, mandibular teeth = salmon, left mandibular fat body = green, left TPC = red.

In spite of this long history of research, the structure/function complex that is the odontocete hearing apparatus is still poorly understood. Over the past forty years there has been general agreement that sound enters the dolphin's head through the “acoustic window”, a thinned portion of the posterior mandible (Figure 1), and is transmitted via the mandibular fat body (MFB) to the bony tympanoperiotic complex [8]. In the time since Norris' seminal paper was published, several studies have produced evidence that sound also enters the MFB via a “gular pathway”, i.e., through the soft tissues around the tongue and throat, eventually passing through the opening created by the absence of the medial bony wall of the posterior mandible [8], [15]–[19]. Discussions continue as to whether the middle ear with its specialized ossicular triumvirate also functions in odontocete hearing [13], [20]. Different explanations for the transfer of sound energy to the inner ear have been offered (for a review, [21]).

The focus of this paper is functional in nature. Traditional anatomic methods and technologically sophisticated techniques allowed us to piece together this puzzling part of the odontocete sound reception apparatus. Sound is “received” over the surface of the animal's head, entering channels (Figures 1, 2, 3, 4 and 5) that eventually lead to the bony ear complex (Figures 6, 7, 8, 9, 10, 11, 12, 13 and 14). The sound reception apparatus, or peripheral auditory system, is comprised of an intricate set of structures that includes fat pads and channels; thin and thicker wafers of dense bone, sheets of connective tissue; along with the requisite muscles, innervations, and vasculature. We assert that, by various means, this structural amalgamation filters and transmits selective acoustic frequencies to the tympanoperiotic complex (TPC), which contains the inner ear of the cochlea (Figure 12). This study of the sound reception system collected data using multiple methods, such as hand dissection, remote imaging followed by digital image-processing, and functional analysis using finite element modeling simulations. We also present a unique analysis of the vibratory complexity of the bony TPC (Figures 15, 16, 17, 18, 19, 20, 21, 22, 23, 24, 25, 26, 27, 28, 29, 30, 31, 32, 33, 34, 35, 36 and 37).

**Figure 2:**
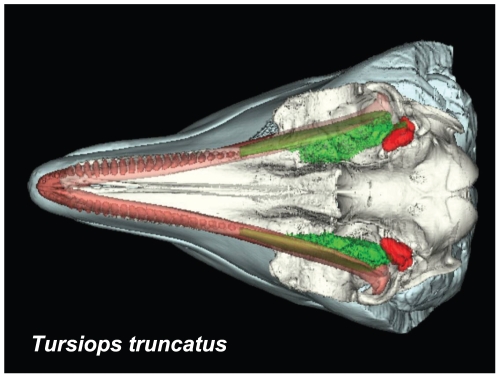
Ventral view of the sound reception anatomy in *Tursiops truncatus*, reconstructed from CT scans. Skin = cyan, skull = ivory, teeth and mandibles = salmon, mandibular teeth = salmon, mandibular fat bodies = green, TPC's = red.

**Figure 3:**
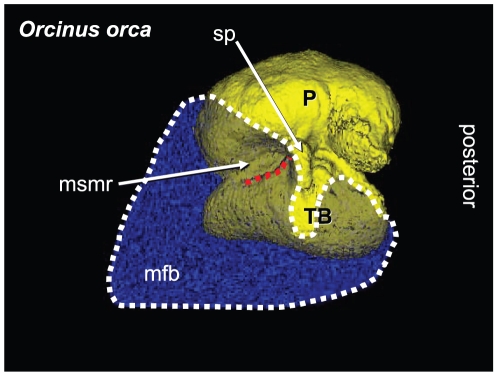
Lateral view of the left TPC and corresponding mandibular fat body (MFB) in *Orcinus orca*. This volume has been reconstructed from CT scans of a Killer Whale (*Orcinus orca*) from the region around the TPC (0.3662 mm cubic voxels). As a consequence, the anterior boundary of the MFB has been artificially terminated at the anterior limit of the scanned volume. The entire head of this specimen was scanned and, as in all other odontocetes in this study, the MFB is continuous from the enlarged foramen of the mandible to its bifurcated attachment to the TPC (shown in this figure). The mandibular fat body is displayed as semi-transparent (blue), outlined in white dots, and overlies the TPC (yellow). The mallear ridge is indicated by the red dotted line. Other structures are as follows: P = periotic bone; TB = tympanic bulla; sp = sigmoid process; msmr = medial sulcus of the mallear ridge (bony funnel); mfb = mandibular fat body. The ventral branch of the MFB attaches to the tympanic bulla and the dorsal branch fits into the medial sulcus of the mallear ridge.

**Figure 4:**
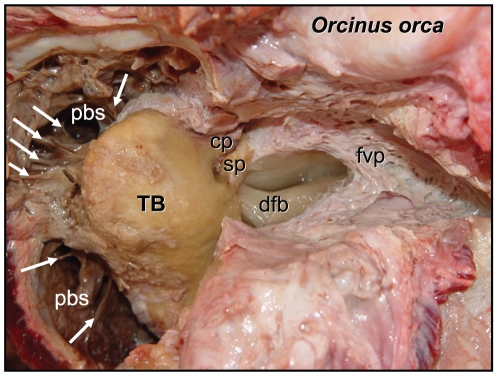
Posteroventral view of the anatomy around the right TPC in *Orcinus orca*, from hand dissection. TB = tympanic bulla, pbs = peribullary sinus, cp = conical process of TPC, sp = sigmoid process of TPC, fvp = fibrous venous plexus, dfb = dorsal branch of the mandibular fat body. The white arrows point to fibers that tether the periotic bone to the periotic fossa of the basicranium (skull).

**Figure 5:**
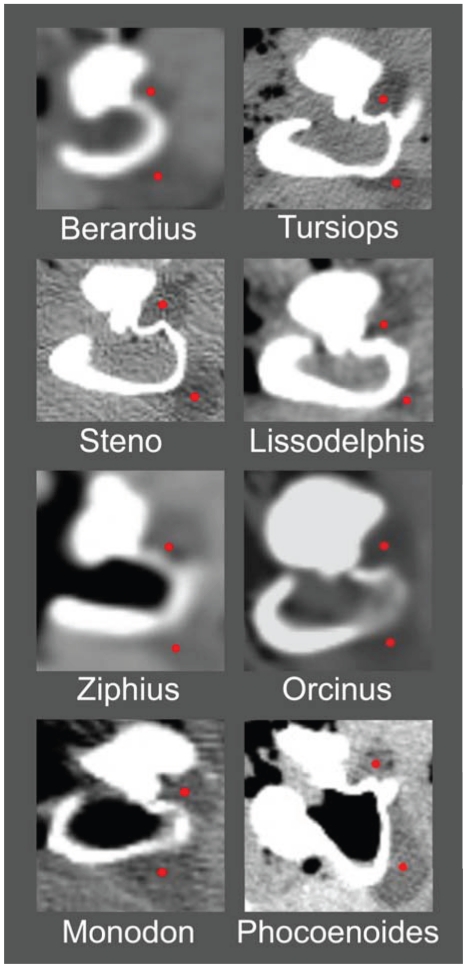
Examples showing the bifurcated mandibular fat body (indicated by red dots) attached to the TPC. Each panel shows a transverse section, from CT scans, through the TPC (white), and both branches of the mandibular fat body (dark gray marked with red dots). Note that the lower branch of the MFB (lower red dot in each subpanel) attaches on the tympanic bulla. The upper branch of the MFB (upper red dot in each subpanel) fits into the funnel or notch between the tympanic and periotic bones. These examples span all major groups of odontocetes except sperm whales (Physeteroidae) and the eclectic “river dolphins” (Pontoporiidae + Platanistoidae). The panels are not scaled equally.

**Figure 6:**
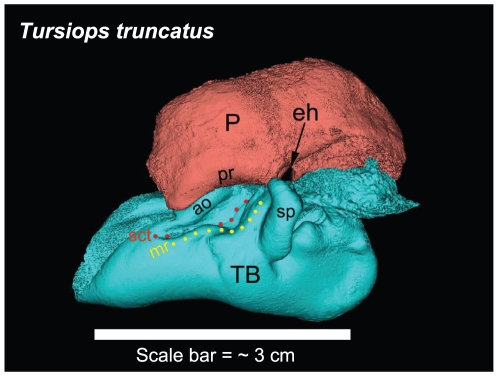
Lateral view of the left TPC from *Tursiops truncatus* reconstructed from micro-CT scans, showing major landmarks. P = periotic bone; TB = tympanic bulla; eh = epitympanic hiatus; pr = parabullary ridge; ao = accessory ossicle; sp = sigmoid process; mr = mallear ridge (yellow dots); sct = sulcus for the chorda tympani (red dots). The scale bar represents approximately 3 cm. It is meant to give the reader an impression of the approximate size of the TPC and is not to be used to measure from point to point, considering that this is 3-D topography projected onto a 2-D plane. All TPC images of *Tursiops truncatus* shown in this report were reconstructed from micro-CT scans (45 micron cubic voxels).

**Figure 7:**
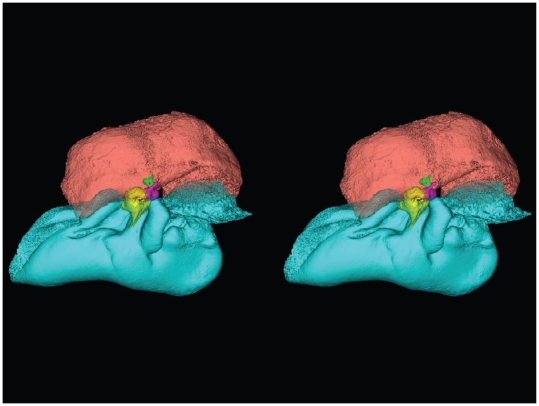
Stereogram of lateral view of the TPC from *Tursiops truncatus*. The images were constructed using transparency to show the ossicles of the middle ear. The periotic bone is salmon colored, the tympanic bone is colored cyan, and the ossicles are colored as follows: malleus = yellow, incus = magenta, stapes = green. (Stereogram viewing instructions: http://www.microscopy-uk.org.uk/mag/indexmag.html?http://www.microscopy-uk.org.uk/mag/artsep00/pjstereo.html).

**Figure 8:**
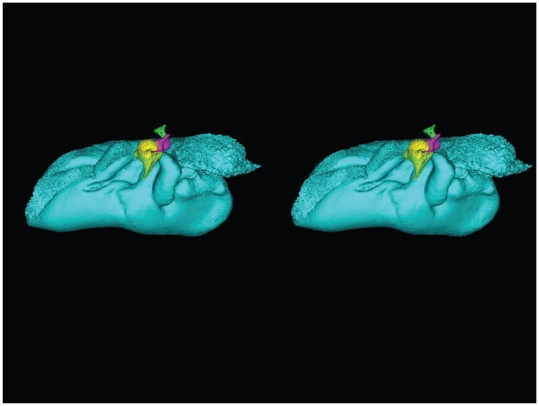
Stereogram of a dorsolateral view of the tympanic bone and ossicles from *Tursiops truncatus*. The images were constructed using transparency to show the relationships between the various bones. The tympanic bone is colored cyan, and the ossicles are colored as follows: malleus = yellow, incus = magenta, stapes = green.

**Figure 9:**
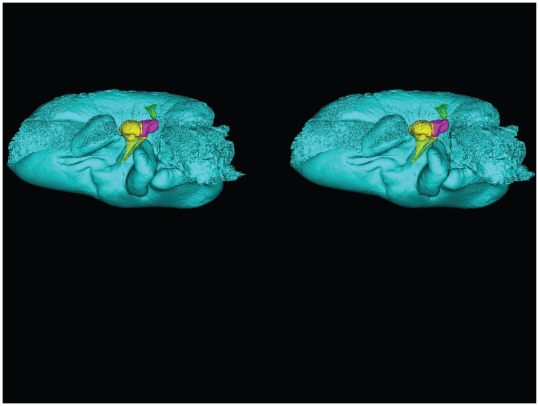
Stereogram of a dorsal view of the tympanic bone and ossicles from *Tursiops truncatus*. The images were constructed using transparency to show the relationships between the bones. The tympanic bone is colored cyan, and the ossicles are colored as follows: malleus = yellow, incus = magenta, stapes = green. This view is a particularly good vantage point from which to view the structures that receive the dorsal branch of the mandibular fat body.

**Figure 10:**
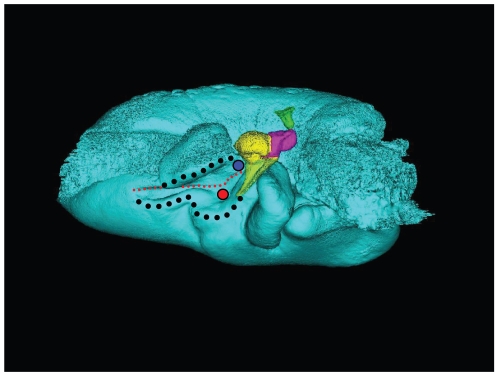
Dorsal view of the tympanic bone and ossicles from *Tursiops truncatus*. This view is also a particularly good vantage point from which to view the structures that receive the dorsal branch of the mandibular fat body. Subtle, but potentially important, landmarks are: two very thin bony locations marked by the prominent red and blue circles. The S-shaped black dotted-line marks the mallear ridge. The shorter black dotted line marks the ridge along the accessory ossicle. The small red dotted-line marks the sulcus for the chorda tympani. The tympanic bone is colored cyan, and the ossicles are colored as follows: malleus = yellow, incus = magenta, stapes = green.

**Figure 11:**
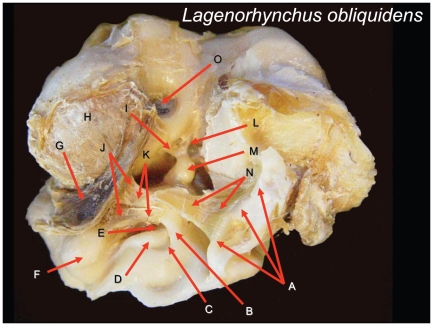
Ventromedial view of the left tympanoperiotic complex from a Pacific White-sided Dolphin (*Lagenorhynchus obliquidens*). The tympanic bulla has been removed for easy viewing of the structures within the tympanic cavity. Structures are as follows: (A) Sigmoid process (the inside of a hollow tube); (B) Anterior (gracile) process of malleus; (C) Thinnest bony window of the medial sulcus of mallear ridge; (D) Keel of the medial sulcus of mallear ridge; (E) Thin window of the medial sulcus of mallear ridge; (F) Accessory ossicle (*processus tubarius*); (G) Desiccated portion of fibrous venous plexus; (H) *Pars cochlearis*; (I) Stapes; (J and K) Anterior ligament of the malleus; (L) Stapedial muscle; (M) Incus; (N) Tympanic ligament (homologous to the ancient tympanic membrane) where it attaches to the sigmoid process, a portion of the tympanic ring; (O) Round window (wherever possible the terminology follows Mead and Fordyce [37]).

**Figure 12:**
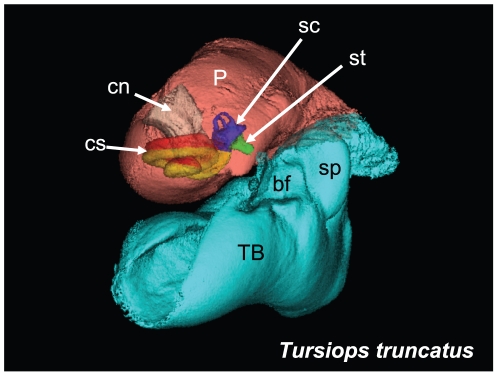
Anterior view of the left TPC from the second specimen of *Tursiops truncatus*. The accessory ossicle of the tympanic bone (TB) has been removed to more clearly demonstrate the relative position of the cochlear spiral (cs), the semicircular canals (sc), and the cochlear nerve (cn) or eighth nerve, all contained within the periotic (P) bone. The *scala vestibuli* (yellow) and *scala tympani* (red), components of the cochlear spiral, are shown in relationship to the semicircular canals (blue), the stapes (green), the (TB) tympanic bone (cyan) and the (P) periotic (salmon). Careful inspection of the stapes (st), near the tip of the white arrow, reveals a small dimple that represents the stapedial foramen. The floor of the medial sulcus of the mallear ridge, the “ear trumpet” or “bony funnel” (bf), which receives the (cone-shaped) dorsal branch of the mandibular fat body, is also shown in this view.

**Figure 13:**
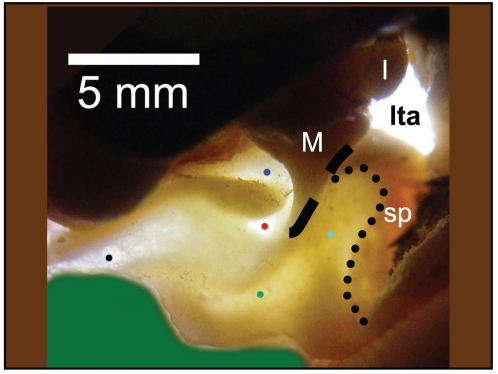
A view from inside the tympanic cavity of *Lagenorhynchus obliquidens*. This is a backlit view through the translucent floor of the ear trumpet (sulcus of mallear ridge). M = malleus; I = incus; sp = sigmoid process; lta = lower tympanic aperture. The black dotted line indicates the anterior border of the sigmoid process. The black dashed line represents the ankylosed (fused) border between the malleus and the tympanic bone. The various colored dots represent locations where the thickness of the bone was measured. Red = 0.26 mm, Blue = 0.36 mm, Black = 0.43 mm; Green = 0.79 mm; Cyan = 0.89 mm. The scale bar represents 5 mm.

**Figure 14:**
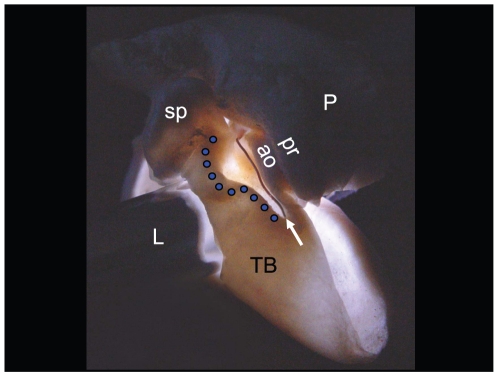
Backlit view of the right TPC from *Lagenorhynchus obliquidens*. The bright field in the center of the image shows the thin, funnel-shaped bony receptacle that receives the dorsal branch of the mandibular fat body, and together form the “ear trumpet.” The funnel resembles a river valley that is bounded by the mallear ridge (blue dots) laterally and the accessory ossicle (ao) medially. This unique view was created by inserting a light source (L) into a hole in the lateral wall of the tympanic bulla (TB) just below the sigmoid process (sp). The blue dots trace the course of the mallear ridge. The white arrow points to a piece of copper wire (0.23 mm diameter) used to indicate the approximate path of the sulcus for the chorda tympani (a nerve that branches off of the facial nerve, Cranial Nerve VII). P = periotic bone; pr = parabullary ridge of the periotic bone.

**Figure 15:**
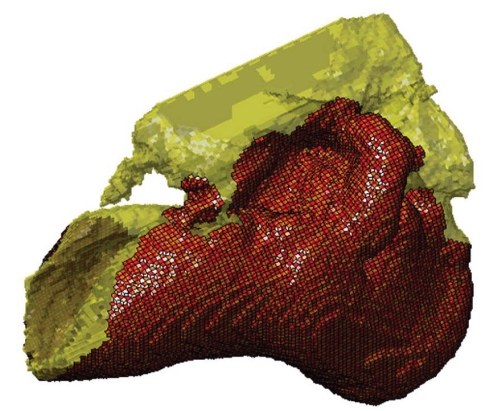
Mesh of the wet boundary layer (red) that approximates soft tissue. The rest of the boundary is either dry or immobile (i.e., parts of the periotic bone) and hence ignored in the wet-mode analysis.

**Figure 16:**
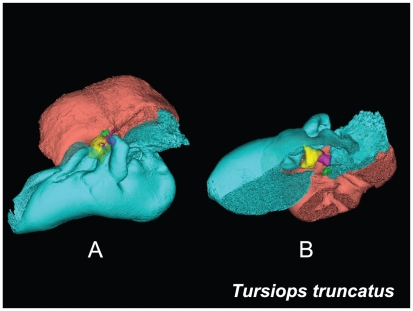
Two views of the left TPC from *Tursiops truncatus*. The left image (A) shows an anterolateral view. The right image (B) is an inverted version of the first image, except that a portion of the medial aspect of the TPC was removed to facilitate viewing the middle ear ossicles. The periotic bone is salmon colored, the tympanic bone is cyan, and the ossicles are as follows: malleus = yellow, incus = magenta, stapes = green.

**Figure 17:**
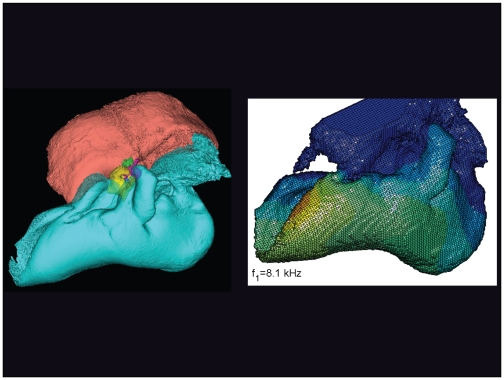
The vibrational pattern representing the 1^st^ natural mode of vibration at 8.1 kHz. The left image, in this and the next seven figures, is a lateral view of the left TPC from *Tursiops truncatus*, reconstructed from micro-CT scans. The periotic bone is salmon colored, the tympanic bone is cyan, and the ossicles are: malleus = yellow, incus = magenta, stapes = green. The image on the right always shows the vibrational pattern, in this case calculated for the first natural mode of vibration at 8.1 kHz. The warm colors indicate the largest displacements of the elements and the cold colors represent the smallest displacements. This figure is linked to the animation sequence that depicts the vibrational mode (Figure S17). The vibrations have been exaggerated for easier viewing (see methods section).

**Figure 18:**
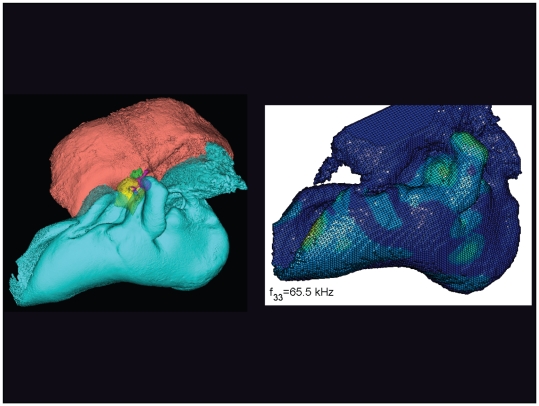
The vibrational pattern representing the 33^rd^ natural mode of vibration at 65.5 kHz. This figure is linked to the animation sequence that depicts the vibrational mode (Figure S18). The vibrations have been exaggerated for easier viewing (see methods section).

**Figure 19:**
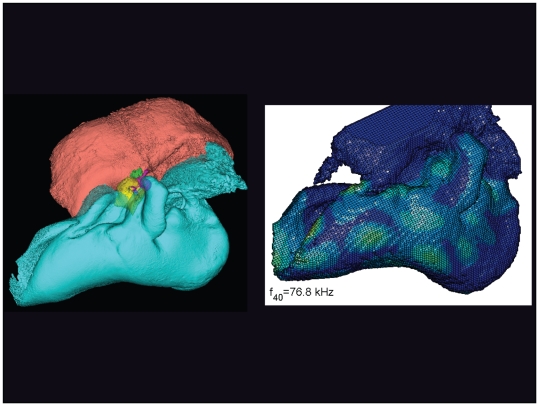
The vibrational pattern representing the 40^th^ natural mode of vibration at 76.8 kHz. This figure is linked to the animation sequence that depicts the vibrational mode (Figure S19). The vibrations have been exaggerated for easier viewing (see methods section).

**Figure 20:**
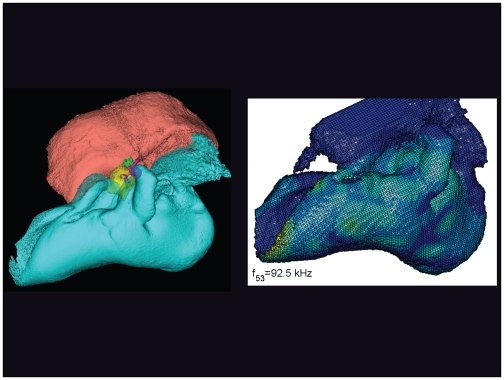
The vibrational pattern representing the 53^rd^ natural mode of vibration at 92.5 kHz. This figure is linked to the animation sequence that depicts the vibrational mode (Figure S20). The vibrations have been exaggerated for easier viewing (see methods section).

**Figure 21:**
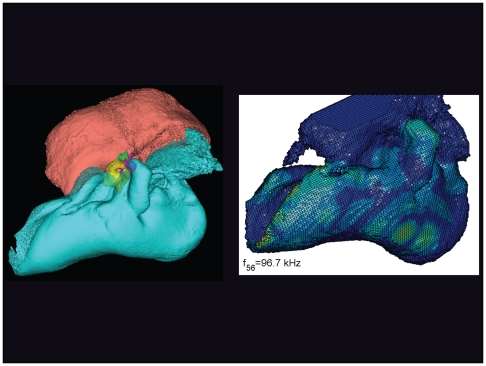
The vibrational pattern representing the 56^th^ natural mode of vibration at 96.7 kHz. This figure is linked to the animation sequence that depicts the vibrational mode (Figure S21). The vibrations have been exaggerated for easier viewing (see methods section).

**Figure 22:**
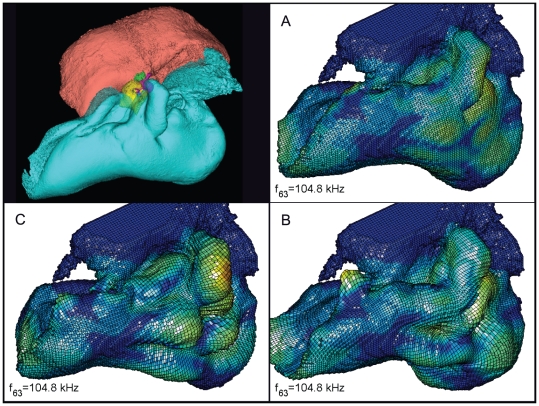
The vibrational pattern representing the 63^rd^ natural mode of vibration at 104.8 kHz. This figure contains three additional panels for the reader that does not have facilities to display the animation sequences or are working from a (motionless) hard copy. The three panels show the two extremes of the oscillation sequence (A and C), as well as the midpoint of the sequence (B). By careful examination and comparison of these three panels, the reader should be able to understand the range of motion in the animation. This figure is linked to the animation sequence that depicts the vibrational mode (Figure S22). The vibrations have been exaggerated for easier viewing (see methods section).

**Figure 23:**
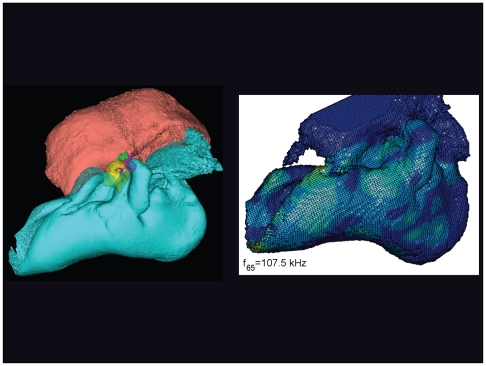
The vibrational pattern representing the 65^th^ natural mode of vibration at 107.5 kHz. This figure is linked to the animation sequence that depicts the vibrational mode (Figure S23). The vibrations have been exaggerated for easier viewing (see methods section).

**Figure 24:**
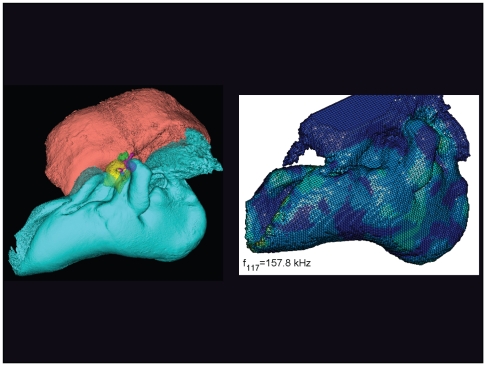
The vibrational pattern representing the 117^th^ natural mode of vibration at 157.8 kHz. This figure is linked to the animation sequence that depicts the vibrational mode (Figure S24). The vibrations have been exaggerated for easier viewing (see methods section).

**Figure 25:**
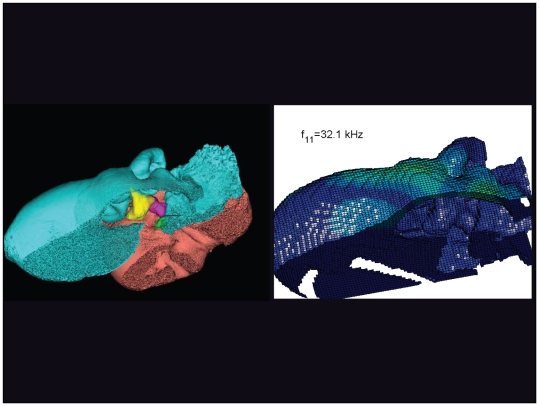
An inverted view of the TPC from *Tursiops truncatus* at 32.1 kHz. The left image, in this and the next ten figures, is an inverted medial view of the left TPC from *Tursiops truncatus*, reconstructed from micro-CT scans. The periotic bone is salmon colored, the tympanic bone is cyan, and the ossicles are: malleus = yellow, incus = magenta, stapes = green. The image on the right always shows the vibrational pattern, in this case calculated for the first natural mode of vibration at 32.1 kHz, the 11^th^ natural mode of vibration. The entire TPC was included during the numerical analysis calculations, but the medial portion was later removed to facilitate viewing the middle ear ossicles. Warm colors indicate the largest displacements of the elements and the cold colors represent the smallest displacements. This figure is linked to the animation sequence that depicts the vibrational mode (Figure S25). The vibrations have been exaggerated for easier viewing (see methods section).

**Figure 26:**
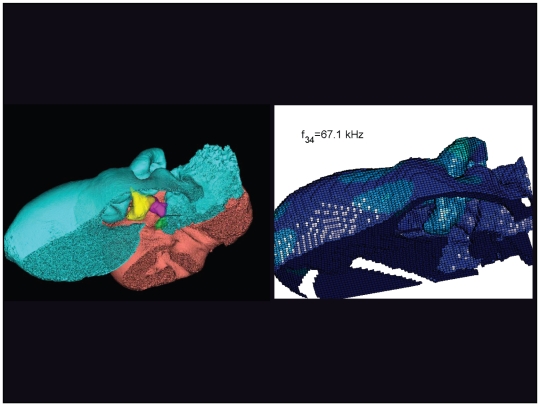
An inverted view of the TPC from *Tursiops truncatus* at 67.1 kHz. The entire TPC was included during the calculation, but the medial portion was removed to facilitate viewing the middle ear ossicles. This simulated vibrational pattern represents the 34^th^ natural mode of vibration. This figure is linked to the animation sequence that depicts the vibrational mode (Figure S26). The vibrations have been exaggerated for easier viewing (see methods section).

**Figure 27:**
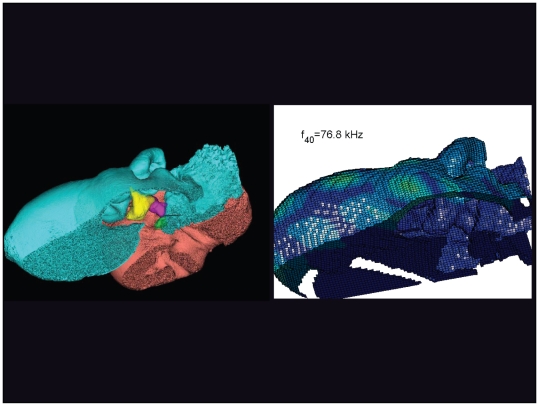
An inverted view of the TPC from *Tursiops truncatus* at 76.8 kHz. The entire TPC was included during the calculation, but the medial portion was removed to facilitate viewing the middle ear ossicles. This simulated vibrational pattern represents the 40^th^ natural mode of vibration. This figure is linked to the animation sequence that depicts the vibrational mode (Figure S27). The vibrations have been exaggerated for easier viewing (see methods section).

**Figure 28:**
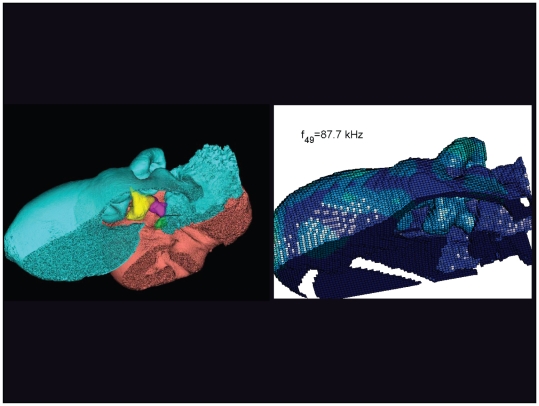
An inverted view of the TPC from *Tursiops truncatus* at 87.7 kHz. The entire TPC was included during the calculation, but the medial portion was removed to facilitate viewing the middle ear ossicles. This simulated vibrational pattern represents the 49^th^ natural mode of vibration. This figure is linked to the animation sequence that depicts the vibrational mode (Figure S28). The vibrations have been exaggerated for easier viewing (see methods section).

**Figure 29:**
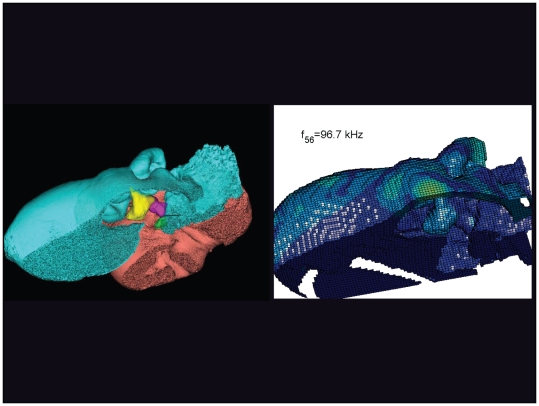
An inverted view of the TPC from *Tursiops truncatus* at 96.7 kHz. The entire TPC was included during the calculation, but the medial portion was removed to facilitate viewing the middle ear ossicles. This simulated vibrational pattern represents the 56^th^ natural mode of vibration. This figure is linked to the animation sequence that depicts the vibrational mode (Figure S29). The vibrations have been exaggerated for easier viewing (see methods section).

**Figure 30:**
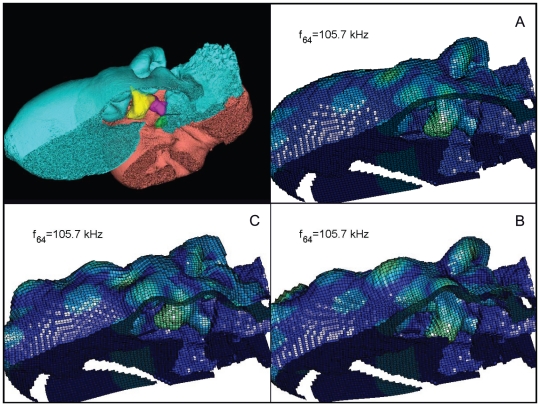
An inverted view of the TPC from *Tursiops truncatus* at 105.7 kHz. This figure contains three additional panels for the reader that does not have facilities to display the animation sequences or are working from a (motionless) hard copy. The three panels show the two extremes of the oscillation sequence (A and C), as well as the midpoint of the sequence (B). By careful examination and comparison of these three panels, the reader should be able to understand the range of motion in the animation. The entire TPC was included during the calculation, but the medial portion was removed to facilitate viewing the middle ear ossicles. This simulated vibrational pattern represents the 64^th^ natural mode of vibration. This figure is linked to the animation sequence that depicts the vibrational mode (Figure S30). The vibrations have been exaggerated for easier viewing (see methods section).

**Figure 31:**
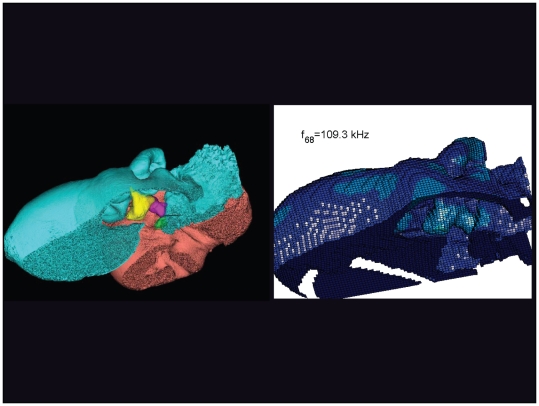
An inverted view of the TPC from *Tursiops truncatus* at 109.3 kHz. The entire TPC was included during the calculation, but the medial portion was removed to facilitate viewing the middle ear ossicles. This simulated vibrational pattern represents the 68^th^ natural mode of vibration. This figure is linked to the animation sequence that depicts the vibrational mode (Figure S31). The vibrations have been exaggerated for easier viewing (see methods section).

**Figure 32:**
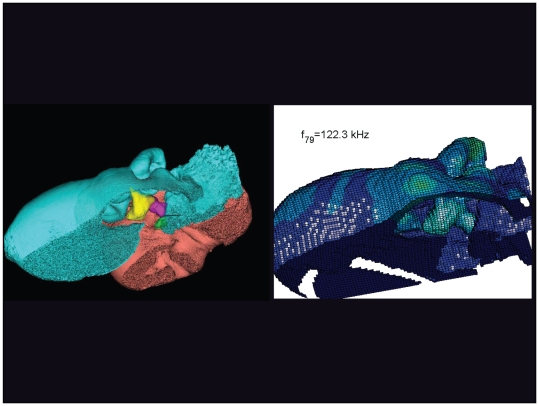
An inverted view of the TPC from *Tursiops truncatus* at 122.3 kHz. The entire TPC was included during the calculation, but the medial portion was removed to facilitate viewing the middle ear ossicles. This simulated vibrational pattern represents the 79^th^ natural mode of vibration. This figure is linked to the animation sequence that depicts the vibrational mode (Figure S32). The vibrations have been exaggerated for easier viewing (see methods section).

**Figure 33:**
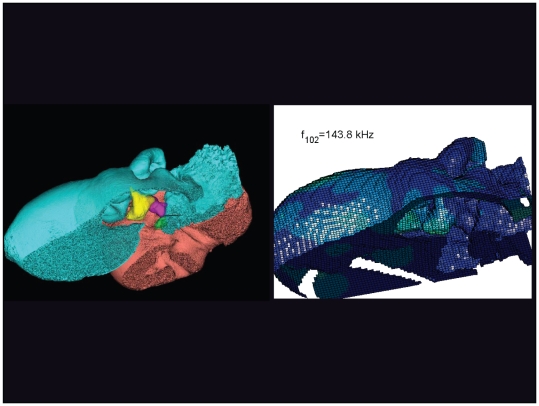
An inverted view of the TPC from *Tursiops truncatus* at 143.8 kHz. The entire TPC was included during the calculation, but the medial portion was removed to facilitate viewing the middle ear ossicles. This simulated vibrational pattern represents the 102^nd^ natural mode of vibration. This figure is linked to the animation sequence that depicts the vibrational mode (Figure S33). The vibrations have been exaggerated for easier viewing (see methods section).

**Figure 34:**
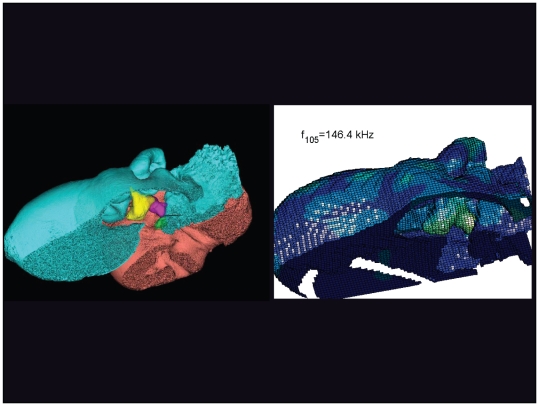
An inverted view of the TPC from *Tursiops truncatus* at 146.4 kHz. The entire TPC was included during the calculation, but the medial portion was removed to facilitate viewing the middle ear ossicles. This simulated vibrational pattern represents the 105^th^ natural mode of vibration. This figure is linked to the animation sequence that depicts the vibrational mode (Figure S34). The vibrations have been exaggerated for easier viewing (see methods section).

**Figure 35:**
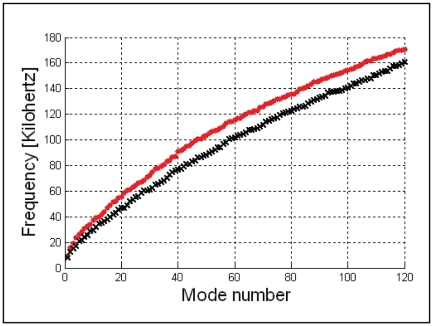
The difference between the “wet” (black crosses) and “dry” (red circles) vibrational modes. These differences span the range of frequencies that were calculated for the first 120 modes. It shows that adding a layer of soft tissue to the lateral aspect of the TPC (wet) causes a slight shift in the frequency.

**Figure 36:**
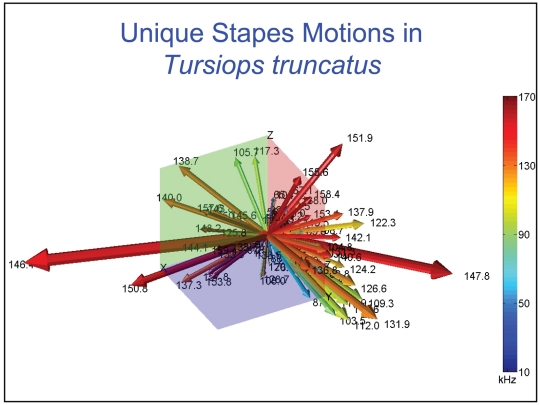
Unique motions of the head of the stapes in the Atlantic Bottlenose Dolphin (*Tursiops truncatus*). Vector arrows show the relative magnitude and direction of motions at the head of the stapes for each natural mode of vibration or resonant frequency (the frequency is indicated by the numbers at each arrowhead). The Z axis runs through the head of the stapes and is more or less perpendicular to the footplate, which is in the XY plane. Colors code for frequencies: blue is low-frequency (on the order of 10 kHz), and red is high-frequency (∼60 kHz). Numbers associated with the arrows indicate the corresponding frequency. The vector arrows demonstrate that the complex vibrational patterns are unique for each natural mode of vibration and may be uniquely coded in the inner ear.

**Figure 37:**
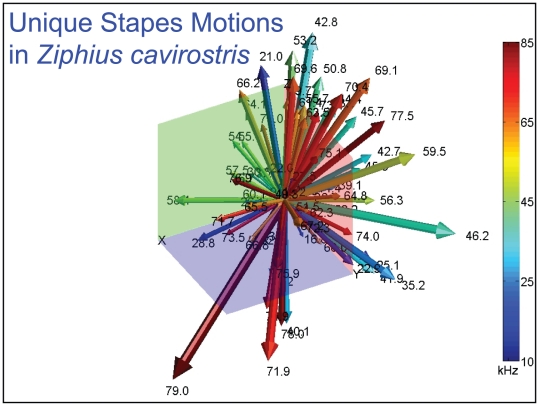
Unique motions of the head of the stapes in Cuvier's Beaked Whale (*Ziphius cavirostris*). Vector arrows show the relative magnitude and direction of motions at the head of the stapes for each natural mode of vibration or resonant frequency (the frequency is indicated by the numbers at each arrowhead). The Z axis runs through the head of the stapes and is more or less perpendicular to the footplate, which is in the XY plane. Colors code for frequencies: cold colors are low-frequency and the warm colors are high-frequency. The numbers associated with the arrows indicate the corresponding frequency. Vibrational analysis produces results from calculations based on structure.

### Middle Ear Function

McCormick [22] studied hearing in the bottlenose dolphin (*Tursiops truncatus*) by measuring cochlear potentials in a sedated dolphin exposed to sound. They approached the ear surgically and found that immobilizing the ossicular chain by applying tension to the tympanic ligament attenuated the cochlear potentials by 18 dB with respect to the level obtained during preliminary tests, whereas removing the malleus had a less appreciable effect, reducing the cochlear potential by 4 dB. McCormick and colleagues therefore concluded that the ossicular chain was not involved in the transmission of sound to the cochlea. From these results they suggested that hearing in the bottlenose dolphin occurred via bone conduction.

In response, Fleischer [23] contended that the surgical procedures used by McCormick et al. [22] and their removal of the malleus may have by-passed the ossicular function, particularly if the highly vascular corpus cavernosum was bleeding into the middle ear space (tympanic cavity). In that case, Fleischer surmised that vibrations must have been transmitted through the blood that was released from the corpus cavernosum to the ossicular chain and/or the oval window (*fenestra ovalis*).

In a follow-up, McCormick et al. [24] elaborated upon their bone conduction hypothesis, explaining how it might function to transduce acoustic signals to the cochlea. They described two modes, the *compressional mode* of bone conduction ([25] cited in [24]) and the *translatory mode* of bone conduction ([26] cited in [24]).

Norris (p. 214, [19]) challenged the bone conduction notion because it would not provide differential frequency information or cues for directional discrimination. He also believed that nature would not evolve elaborate ossicular specializations for high-frequency (HF) hearing only to abandon them. The notion that there are two mechanisms for stimulating the inner ear (bone conduction *and* ossicular chain vibration) has not been proposed to function together, albeit for different parts of the frequency spectrum. We will test this idea using the methods presented here.

Based on dissections of the hearing apparatus and mechanical modeling, Fleischer [27] came to the conclusion that the middle ear ossicles must be involved in the transmission of sound to the oval window and the cochlea. His viewpoint was that the tympanic membrane, in combination with a thin bony region of the dorsolateral part of the tympanic bone (a region he dubbed the “tympanic plate”) constituted the sound receiving area, and that vibrations reach the malleus through the tympanic ligament. He also noted that the middle ear bones are specialized for high-frequency hearing, as compared to terrestrial mammals. For example, the middle ear bones are denser and more rigidly connected to one another than in terrestrial mammals. In addition, the muscles attached to the ossicular chain (e.g., stapedial muscle and tensor tympani) are more powerful than they are in similarly sized terrestrial mammals, indicating a well-developed middle ear reflex (Figure 11).

In 2000, Ketten (pp. 73–77, [13]) also reviewed the debate on ossicular chain function. She concluded that middle ear functions were unresolved for all cetaceans ([13], pp. 76–77).

In 1999, Hemilä, Nummela, and colleagues produced a series of three studies [21], [28], [29] offering support for Fleischer's conclusion that the middle ear bones play an active role in sound transduction. They presented another explanation for how the ossicular chain might mechanically transmit sound energy. They also redefined Fleischer's “tympanic plate” to include a significantly larger region of the tympanic bone. They proposed that differential bending motions are initiated by sound that is incident upon their enlarged definition of the tympanic plate. According to their proposal, the vibrations are transmitted to the malleus via the thinly folded sheets of bone at the tympanoperiotic junction, with little more specification. They also suggested that the motion of the malleus was along a single longitudinal axis of the anterior process (*processus gracilis*). While we agree with some of their conclusions, we will present evidence that, in some cases contrasts with, and in other cases elaborates on the models devised by Hemilä, Nummela, and colleagues.

The current paper addresses several questions related to the odontocete hearing structure/function complex: 1) What are the probable sites and mechanisms for acoustic stimulation of the TPC? 2) Is the ossicular chain functional? 3) If so, how might sound pressure be transmitted through the TPC to the cochlea as vibrational motions or displacements?

Answering these questions required us to study the details of the attachments between the mandibular fat body (MFB) and the bony tympanoperiotic complex (TPC) from a comparative viewpoint, to test models that allowed simulations of the functional morphology, and consider possible functional implications.

## Materials and Methods

### Ethics Statement

Postmortem toothed whale specimens for this project were obtained from five sources: National Marine Fisheries Service (NMFS), Navy Marine Mammal Program (NMMP), SeaWorld San Diego, Portland State University, and the National Museum of Natural History at the Smithsonian Institution. Dr. Cranford has an Authorization Letter from the NMFS to possess marine mammal specimens for research purposes. The San Diego State University Institutional Animal Care and Use Committee (IACUC) has reviewed and approved our methods for handling, dissecting, and disposal of postmortem marine mammal tissue samples. Their approval was issued in a document (APF# 09-05-014B) entitled, “Marine Mammal Dissections” and is dated 17 June 2009.

### Gross Morphology of the peripheral auditory system – “skin to TPC” – (gleaned from physical specimens and remote imaging)

Over the past twenty years, one of us (Cranford) has studied x-ray computed tomography (CT) scans from more than 30 species of odontocetes [30]–[32]. All specimens in this report were subjected to CT scanning. The large size of the specimens and advancements in CT technology has dictated that the exact imaging parameters for specimens have necessarily changed over the years. The parameters employed herein were always sufficient to discriminate the smallest structures of interest.

Interpreting or “reading” CT scans requires anatomic experience from dissections and repeated exposure to a variety of image sets. Interpretations of images are particularly difficult for species that have not been scanned previously or for which there is little anatomic data (Cranford et al. [18]). Two of us (Cranford and Amundin [33]) have conducted dissections on a few dozen odontocetes species and have become familiar with the details of the anatomy, particularly the tissue geometry, and the interfaces between various structures.

For this study, we examined the gross morphology of the gular anatomy and the MFB in more than 25 odontocete species using hand dissection and remote imaging techniques like CT and MR scanning. The results for 9 of those species are reported here: five delphinids (modern dolphins), one monodontid (allied to dolphins and porpoises), one phocoenid (porpoise), and two ziphiids (beaked whales). Specifically, they are: Atlantic Bottlenose Dolphin (*Tursiops truncatus*), Pacific White-sided Dolphin (*Lagenorhynchus obliquidens*), Killer Whale (*Orcinus orca*), Rough Toothed Dolphin (*Steno bredanensis*), Northern Right Whale Dolphin (*Lissodelphis borealis*), Narwhal (*Monodon monoceros*), Dall's Porpoise (*Phocoenoides dalli*), Cuvier's Beaked Whale (*Ziphius cavirostris*), and Baird's Beaked Whale (*Berardius bairdii*) (Figures 1, 2, 3, 4, 5, 6, 7, 8, 9, 10, 11, 12, 13 and 14).

The gross morphology of the tympanoperiotic complex (a combination of the tympanic bone, ossicles, and periotic bone) was investigated by multiple methods. In addition, a single tympanoperiotic complex from two Atlantic Bottlenose Dolphins (*Tursiops truncatus*) and both TPC's from a single Pacific White-sided Dolphin (*Lagenorhynchus obliquidens*) were extracted, preserved, and scanned with micro-CT.

The left TPC from the *Lagenorhynchus obliquidens* specimen was set aside and the tympanic bulla separated from the periotic with a high-speed dental drill (Figure 11). This provided an unobstructed view of the ossicular chain and associated anatomy in this desiccated specimen. The right TPC from the Pacific White-sided Dolphin already had a small window broken out of the lateral wall of the tympanic bone similar to that illustrated in McCormick et al. [22] (their Fig. 4, p. 1423). This sample provided an opportunity to insert a bright light source (Figure 14) and observe the varied thicknesses of the tympanic bone (Figures 10, 11, 12, 13 and 14), a method also used by Nummela and her colleagues [29].

The gross morphology of the TPC in *Tursiops truncatus* was gleaned from two specimens. One intact left TPC specimen was scanned using micro-CT and the other, also a left TPC, was accidentally fractured and was studied under a dissecting microscope. The intact TPC from *Tursiops truncatus* was subjected to micro-CT scanning using a General Electric CT eXplore Locus in vivo Micro-CT scanner. We CT scanned the *Tursiops truncatus* TPC twice, once into 45 µm slices and again using 27 µm increments (contiguous, cubic voxels without intervening gaps). All of the images and models generated in this paper used the 45 µm cubic voxel data set. Volumetric reconstruction, segmentation, and scientific visualization of all scan data presented here was accomplished using Analyze 9.0 (Mayo) software (Figures 1, 2, 3, 5, 6, 7, 8, 9, 10, 12, 16, and parts of 17, 18, 19, 20, 21, 22, 23, 24, 25, 26, 27, 28, 29, 30, 31, 32, 33 and 34). In addition, the micro-CT scan volume was subjected to vibrational analysis (Figures 15, 16, 17, 18, 19, 20, 21, 22, 23, 24, 25, 26, 27, 28, 29, 30, 31, 32, 33, 34, 35, 36 and 37 and their accompanying animation sequences).

### Vibrational analysis of the TPC in *Tursiops truncatus*

Any structure, no matter how complex, has an entire family of frequencies at which it will vibrate; the so called *natural modes* of vibration or *resonant frequencies* of vibration. These natural modes were calculated in the vibrational analysis of a (left) TPC from an Atlantic Bottlenose Dolphin, *Tursiops truncatus* (NMMP specimen YOG) scanned 12 March 2005 on a GE Explore Locus Micro-CT scanner.

The original 45 µm CT data translated into an unmanageably large finite element model (FEM) [34], [35]. Therefore, the original CT data were sub-sampled with cubic smoothing to achieve a resolution of 360 µm on a side for all cubic voxels.

The exposed surface of the model was equipped with appropriate boundary conditions, as explained below. The finite element model was derived from the resulting volumetric image by converting each voxel corresponding to dense bone into a finite element. A moderate amount of Laplacian smoothing was applied both on the surface and in the interior to enhance the smoothness of the boundary surfaces. The resulting surface mesh is shown in Figure 15.

The malleus-periotic joint (approximating a ball and socket) was modeled as fused. In other words, the malleus was considered attached to the periotic bone through the small rounded interface between these two bones. This is an approximation of mechanical stiffness of the actual fibrous joint, which was adopted mainly because the mechanical properties of the joint are unknown and because the structure of the joint indicates that it is probably fairly stiff [27].

The material properties of bone were taken from a paper by Currey [36], a study that included the tympanic bulla of an adult fin whale (*Balaenoptera physalus*): mass density of 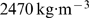, a Young's modulus of 30 GPa, and a Poisson's ratio of 0.2.

The finite element model consisted of 123,000 nodes and 101,000 hexahedral elements. Due to the almost ideal aspect ratio of the elements, the isoparametric formulation of the stiffness was adopted. The mass matrix was taken as diagonal (Hinton, Rock, Zienkiewicz lumping) [37]. Please see Appendix S1 for more details on the formulation of the model.

The periotic bone is relatively stiff and is suspended from and tethered to the skull by a branching network of connective tissue fibers (Figure 4). The fibers originate over the surface of the periotic fossa of the skull and insert upon the periotic bone in several locations. The result is a stiff connection between the skull and TPC by a multitude of fibrous connections, which maintain acoustic isolation. As a consequence, the periotic bone contributes little to the natural vibration modes. Therefore, we trimmed off a large part of the periotic bone from the modeled geometry for gains in efficiency (fewer elements require fewer computations). A no-motion boundary condition was applied on the planar cuts of the periotic bone (Figure 15), which separated the modeled tympanic bone with the ossicles and parts of the periotic bone from the un-modeled bulk of the periotic bone.

First, the *in vacuo* free-vibration modes of the “dry” bone were computed with Matlab's implicitly re-started Arnoldi eigenvalue solver. Next, the contact between the TPC and soft tissues, such as acoustic fats of the MFB, was accounted for. As a first approximation, the tissue was considered to be acoustically equivalent to an incompressible inviscid liquid, which was approximated as infinite in extent. This part of the boundary surface of the TPC was considered “wetted” and is shown in Figure 15. Note that this configuration closely approximates the anatomy shown in Figure 3 for the largest dolphin, the Killer Whale (*Orcinus orca*). In Figure 15, note that parts of the surface of the periotic bone were ignored in the wetted analysis because they were practically immobile. The material properties for the surrounding fluid mimic those of an acoustic fat: mass density of 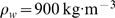, and the speed of sound 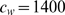 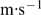.

The formulation adopted was based on Antoniadis and Kanarachos [38]. First, a set of modal vectors for the structural displacements, the corresponding modal mass, and stiffness matrix were computed. Then, using the modal basis vectors as the normal pressure derivative (Neumann), boundary conditions on the fluid-structure interface result in a set of potential (Laplacian) problems for the pressure modes in the fluid. Finally, the solutions for these pressure modes were used to derive the added-mass matrix, and a modified reduced eigenvalue problem was solved to compute the wetted free-vibration frequencies and the mixture coefficients for the *in vacuo* free-vibration modes to yield the wetted modal vectors. The potential problems were solved with a simple boundary element program based on a piece-wise “constant” approximation on the quadrilateral surface mesh. Nevertheless, the potential problems required substantial computations due to the relatively large number of unknowns (over 21,000). The general effect observed with the addition of the wetting was a lowering of the natural frequencies (on the order of 10 to 15%) and a relatively insignificant mixing of adjacent *in vacuo* natural modes together to form the wetted vibration modes (Figure 35).

Additional simulations were also run to check the possible effects of trimming the periotic on the simulation results. For the purposes of this test, we used the same CT scan of the TPC from the same specimen of *Tursiops truncatus*. The entire TPC was modeled (not trimmed), and it was assumed to be “free floating” (not anchored). This second model and boundary conditions could be considered more or less opposite to the first specimen, which was trimmed and anchored. The first 100 non-zero *in vacuo* natural modes of vibration were calculated and inspected for both models and their vibrational analysis results were compared.

Supplementary simulations of the free-floating configuration were run to assess the effect of the discretization error (this error is controlled by the resolution of the computational mesh; the finer the mesh, the smaller the discretization error). The simulations were repeated with tetrahedral meshes and an assumed-strain formulation [39], using larger mesh sizes of 480 µm and 720 µm. The relative frequency differences in selected corresponding natural modes, between the hexahedral model using a resolution of 360 µm and the two tetrahedral models (480 µm and 720 µm), were below 10%. In conjunction with the knowledge of the quadratic convergence rate of the frequencies of free vibration, we can estimate that the true errors of the 360 µm model were estimated at <3.3% [40].
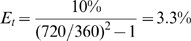



As is well known, the free vibration modes are determined by the vibration analysis only up to an arbitrary scaling factor. In other words, the amplitude of the free vibration shape is arbitrary. Therefore, in the figures used to visualize the free vibration modes (Figures 17, 18, 19, 20, 21, 22, 23, 24, 25, 26, 27, 28, 29, 30, 31, 32, 33 and 34), we present the mode shapes with highly exaggerated amplitude in order to illustrate the distribution of the locations of very small or zero amplitude motion (the so-called vibration nodes) and of the locations of large amplitude motion (the vibration anti-nodes). When the sound pressure forcing and a realistic damping are included in the so-called forced-vibration analysis, the amplitudes of motion may be determined. For the sound pressures of interest we would expect the amplitudes of the vibrating TPC to be on the order of micrometers.

## Results

### Morphology of the Peripheral Auditory System

In recounting these results, we will follow our presumed primary pathway for high-frequency sound reception, from the surface of the head to the footplate of the stapes. This presumptive organization is based on interpretation of the vast literature on odontocete sound reception anatomy and physiology, plus the results of our modeling efforts.

The conventional and widely accepted notion for the primary acoustic pathway into the odontocete head was first described by Norris [8]. It begins at a fatty pad, the “acoustic window,” that bulges between the skin and the lateral surface of the pan bone, which comprises a large portion of the posterior mandibles, giving the outward appearance of a swollen jaw in most odontocetes. In the center of the region where the acoustic window contacts the lateral mandible, the pan bone is so thin as to be translucent and the thickness of the bone varies across the lateral wall [8], [41]. The medial walls of the flared posterior mandibles are absent, a feature common to all extant odontocetes. The origin of this characteristic can be traced back into the fossil record to the earliest archaeocetes [42].

The Norris paradigm served well as a stimulus for testing ideas and comparing results over the past four decades. But Norris himself was under no illusions that his “jaw hearing” hypothesis provided all of the answers. In fact, he predicted that sound enters the odontocete head from multiple locations, presupposed a gular sound reception pathway (for which we now have substantial evidence from numerical analysis and psychoacoustic experiments), and speculated on the functional necessity, and possible characteristics of, an internal acoustic pinna [7], [8], [10], [43].

The posterior third of the odontocete mandible has a greatly enlarged lumen; a hollow that is filled with a peculiar pellucid lipid and connective tissue (Figures 1 and 3), an organ known as the mandibular fat body (MFB). Since the medial wall of the posterior mandible is absent, it leaves an unobstructed pathway or “open door” for the passage of sound from the gular region into the MFB, which bulges medially (Figure 2). The MFB stretches posteriorly from the expansive lumen of the mandible to the bony ear complex or TPC. The MFB extends beyond the mandible posteriorly and tapers in most odontocetes until it forks into a dorsal and a ventral branch, each of which attaches to the TPC (Figure 3). The ventral branch is the larger of the two and is familiar from the literature on odontocete sound reception anatomy [7], [8], [22]. These fatty branches are also ensheathed in connective tissue, part of the fibrous venous plexus (Figure 4).

The larger, ventral branch attaches between the outer lip and the median furrow of the tympanic bulla, along the length of the bulla from the anterodorsal crest to approximately the outer posterior prominence, where the bone of the tympanic bulla becomes greatly thickened [7], [8], [11], [44], [45] (Figure 3).

The small, inconspicuous dorsal branch of the tapered posterior MFB has thus far been largely overlooked, although Ridgway [46] makes a brief mention of it in a contribution honoring the life's work of Kenneth S. Norris. The terminus of the dorsal branch of the MFB forms a cone-shaped fat body that fits into a bony funnel just anterior to the sigmoid process of the TPC (Figure 4); (also see Mead and Fordyce [45], Fig. 25y). This posterior branching pattern and the distinct attachments to the TPC are similar across a broad taxonomic spectrum of odontocetes (Figure 5). This comparative series shows the attachment of the two branches of the MFB onto the tympanic bulla in the two locations, as described. These images are from CT scans of intact specimens. The examples span all major groups of odontocetes except sperm whales (Physeteroidae) and the eclectic “river dolphins” (Pontoporiidae+Platanistoidae) [47].

To our knowledge, the cone-shaped dorsal branch of the MFB and the bony funnel that contains it has no specific moniker, so we have dubbed this structural complex the “ear trumpet” because of its potential to carry sound energy into the TPC. We will explain this decision in greater detail in the following passages.

The bony funnel is formed by portions of the tympanic bone (the sigmoid process, the mallear ridge, the sulcus for the chorda tympani, and the accessory ossicle), as well as the parabullary ridge of the periotic. The dorsal branch of the MFB fills this funnel and is thus in contact with all of these bony components (Figure 3). The lateral view of the TPC and the curious funnel is also shown in Figures 6 and 7, from micro-CT scans of *Tursiops truncatus*.

Conceptually, the topography of this bony funnel is somewhat reminiscent of a conventional “river valley,” where the sulcus for the chorda tympani (*Canaliculus chordae tympani*) represents the course of the “river.” The “valley” floor expands on either side of the “river” until it reaches the base of the bounding “mountains” that define the extent of the valley floor and rise to ridges on either side. Carrying this analogy forward, the mountain ridges are formed by the S-shaped mallear ridge on one (lateral) side of the valley and the, more or less, linear ridge formed by the accessory ossicle on the opposite (medial) side of the valley, plus the small ridgeline along the ventromedial aspect of the parabullary ridge of the periotic. The floor of this conceptual valley or bony funnel is also noteworthy because it contains a series of thin translucent bony regions (Figures 10, 11, 13, and 14), as described below.

A semi-transparent stereo view of the TPC from *Tursiops truncatus* was reconstructed from micro-CT scan sections (Figures 7, 8 and 9). These views show the juxtaposition between the floor of the bony funnel and the gracile process of the malleus, where they are fused. The intricate structure of this region and the juxtaposition of the dorsal branch of the mandibular fat body suggest that it is an important place to focus investigative effort into the functional morphology of sound reception and transduction in odontocetes. The functional significance of this region has been largely overlooked by previous studies, possibly because in most instances the TPC was studied in isolation, extracted from its anatomic context. The functional significance becomes clear when considering that the entire bony funnel is filled with acoustic fat from the small dorsal branch of the mandibular fat body (Figure 3).

The river valley analogy is useful for understanding the general form in this region but it is inappropriate anatomic terminology. Thus, as an anatomic reference, we refer to the “valley floor” as the ***medial sulcus of the mallear ridge***. Structurally, the sulcus of the mallear ridge is reminiscent of a funnel for an ear trumpet, a structural and functional similarity that apparently also occurred to Boenninghaus in 1904 [48], [49] from his studies of the harbor porpoise and the sperm whale. The finite element model reported below produced evidence to suggest that the “ear trumpet” terminology is a fair assessment. Consequently, we will refer to this anatomically complex, and functionally significant region of the bony funnel and the fat body it contains as the *ear trumpet* in odontocete cetaceans.

Thus far, the details of anatomic structure of the TPC have been examined from an external perspective. Now, we will delve into the structural characteristics that can be seen from inside the TPC (Figure 11). Observations within the tympanic cavity were facilitated by applying illumination in various ways (Figures 13 and 14), allowing the discovery of the thickness of the bone in various locations and the geometric relationships between other nearby anatomic components.

For example, Figures 10, 13 and 14 show the distribution of thin translucent patches of bone, which vary in size, shape and thickness, in the ventrolateral wall of the sulcus of the mallear ridge. Figure 13 also shows how the tapered anterior process of the malleus (*processus gracilis*) stretches into the center of the region containing the thinnest membrane-like bony patches. Dial vernier calipers were used to measure bone thickness at the thinnest locations (see caption for Figure 13). Measurements of the two thinnest patches (0.26 mm and 0.36 mm) were those adjacent to the anterior process of the malleus, where the malleus is fused to the underside of the medial sulcus of the mallear ridge.

Careful inspection of the malleus showed that the caudal edge of the anterior process is fused to the tympanic bone by a synarthrosis (also called a true synostosis), a butt joint ([27], p. 31; [22], p. 1423; [50], p. 397). The suture for this joint runs from near the apex of the anterior process to a point approximately two thirds of the distance to the incudomallear joint, as noted in Figure 13 for *Lagenorhynchus obliquidens*. This configuration is very similar to that found in specimens of *Tursiops truncatus*, as shown in the stereo pairs of the TPC reconstructed from micro-CT scans (Figures. 7, 8, 9, and 10). (These stereo pairs can be viewed without any special equipment. Instructions for free-viewing stereo pairs can be found at: http://www.microscopy-uk.org.uk/mag/indexmag.html?http://www.microscopy-uk.org.uk/mag/artsep00/pjstereo.html). The joint between the malleus and the tympanic bulla forms a fracture plane that is commonly exposed when the TPC is accidentally dropped or mishandled. The anterior process of the malleus also forms a shelf (away from the butt joint indicated by the dashed line in Figure 13) that overhangs the two thinnest patches of bone in the medial sulcus of the mallear ridge.

The short distance from the medial sulcus of the mallear ridge, along the ossicular chain, to the oval window of the inner ear is the only morphological description needed to complete our presumptive sound transmission pathway. Despite the research papers that question the function of the ossicular chain in odontocetes [13], [20], [51], we present evidence based on morphology and modeling results suggesting that ossicular motion is integral to the function of the TPC. Here we provide observations that lead to the conclusion that the ossicular chain is fully functional for reasons that will become clear in the discussion. There are a number of papers that have reported the detailed anatomy of the individual ossicles, and this study has little to add to those descriptions [45]. We can, however, provide some anatomic context to the ossicles (Figures 7, 8, 9, 10, 11, 12 and 13). Our comments, descriptions, and figures concerning the ossicles are offered primarily in the interest of anatomic geometry, completeness, and as groundwork for the modeling results to follow.

Figures 7, 8, 9 and 10 show the 3-D relationship between the ossicles and the bony parts of the ear trumpet, the funnel that contains the medial sulcus of the mallear ridge. This sequence of figures contains three stereo pairs.

In Figure 11, most of the tympanic bulla has been removed to reveal the anatomy within the tympanic cavity of the left TPC in *Lagenorhynchus obliquidens*. The figure shows the middle ear ossicles partly exposed, as well as a few of the connections to, and in the context of, nearby anatomic components.

Figure 12 shows an anterior view of the left TPC from *Tursiops truncatus*. It demonstrates the relative position of the cochlear spiral, the semicircular canals, the stapes, tympanic and periotic bones.

### Numerical Analysis of the Tympanoperiotic Complex (TPC)

We calculated the first 120 “natural modes” of vibration or “resonant frequencies” of vibration between 8.1 kHz (f_1_) and 160.9 kHz (f_120_).

The most prominent results from examining these natural modes of vibration of the TPC from a *Tursiops truncatus* are:

1. The modes are complex, probably too complex to be ascertained by inspection or, by implication, an *ad hoc* lumped parameter model.
2. The modes change dramatically over the resonant frequency range (Figures 19, 20, 21, 22, 23, 24, 25, 26, 27, 28, 29, 30, 31, 32, 33 and 34).
3. The action of the sulcus of the mallear ridge (and of the connected ossicles) changes with frequency, as do the interactions with other portions of the TPC; e.g., the sigmoid process. Please examine the animation sequences linked to Figures 19, 20, 21, 22, 23, 24, 25, 26, 27, 28, 29, 30, 31, 32, 33 and 34.
4. The motions of the ossicular chain are small or imperceptible at the first six resonant frequencies (f_1_ = 8.1 kHz−f_6_ = 21.8 kHz), and generally become prominent in many higher modes.
5. The motion of the stapes, in particular, is not simply piston-like but exhibits a variety of complex rocking and piston motions across the spectrum of resonant frequencies that were calculated (Figures 36 and 37).

The complete TPC from the same specimen of the Atlantic Bottlenose Dolphin (*Tursiops truncatus*) was modeled a second time in order to assess the possible effects of trimming the periotic bone as described in the Materials and Methods section. The entire TPC was modeled (not trimmed), and it was assumed to be “free-floating” (not anchored). The resolution of the second model was also 360 µm, the same as in the initial simulation for the trimmed TPC. The second model and boundary conditions could be considered an opposite extreme to the first model, which was trimmed and anchored. Selected *in vacuo* natural modes of vibration from the first simulation, which are within the range of best hearing sensitivity of the dolphin, were matched to corresponding modes in the second simulation. Despite the dramatic differences in boundary conditions, the corresponding frequencies differed little (mean relative difference was 5.6%; see Table 1). This suggests that the structural similarity between the two models causes them to respond similarly, even for very different modeling parameters.

**Table 1:** Comparison of frequencies associated with selected modes for two models of the TPC in *Tursiops truncatus*.

Mode	Trimmed, fixed periotic bone	Complete TPC, Free-floating
8	32.41 kHz	33.56 kHz
21	48.95 kHz	45.76 kHz
34	69.62 kHz	62.14 kHz
36	82.89 kHz	78.08 kHz
43	94.69 kHz	96.40 kHz

## Discussion

### Branching of the Posterior Mandibular Fat Body (MFB)

A portion of the advances in our knowledge reported here are based upon complementary investigative techniques, macro and micro CT scanning, hand dissection, and vibroacoustic modeling. The micro and macro CT scans provide unique, undisturbed views of the gross anatomy and geometry of the head and the details of the intricate *in situ* anatomic context in and around the tympanoperiotic complex. The interpretations of the anatomic results from these digital remote images were also verified with traditional hand dissections. The methodological combination of CT scanning and hand dissection has allowed us to uncover the dual fatty pathways into the TPC, pathways that are apparently ubiquitous across the Odontoceti (Figures 1, 2, 3, 4 and 5).

In the scans showing the anatomic context around the odontocete TPC (Figure 5), the dorsal branch of the MFB fills the bony funnel and is in contact with all of its bony components, including the thinnest patches of the tympanic bone and a thick portion of the dense periotic bone, specifically the parabullary ridge of the periotic. In a brief note, Ridgway ([46], p. 929) reported that the mandibular fat body touches two bones of the TPC. Using a uniquely informative image of a section that cut through this region in a bottlenose dolphin, Ridgway noted, “It is apparent from these photographs that the fat body of the lower jaw contacts the auditory bulla and the periotic bone containing the cochlea (tympano-periotic complex).”

We surmise that the contact between the mandibular fat body and the parabullary ridge of the periotic has little or no “sensory” implications. There are two reasons for this supposition. First, the impedance mismatch between the fat body and the thick dense bone comprising the parabullary ridge of the periotic would prevent much acoustic energy from traversing that interface. Second, the relatively large mass of the periotic bone compared to the thin bony interface between the tympanic and the dorsal branch of the mandibular fat body would require concomitantly large forces to cause any displacement in this thickened region of the periotic.

Morphologically, the fatty branches of the MFB indicate that there are at least two acoustic pathways from the surface of the odontocete head to the tympanoperiotic complex. In fact, the acoustic environment within the head of an odontocete undoubtedly contains many pathways (multipaths) through various tissue types that may eventually reach the TPC. The key distinction here is that the pathways we have described are those that the simulations suggest have the potential to carry the largest fraction of acoustic energy to the TPC from that which is incident upon the dolphin's head.

The macro CT scans show that the branching pattern of the posterior mandibular fat body, just before it attaches to the TPC, is typical for odontocetes (Figures 3 and 5), with the possible exception of sperm whales and “river” dolphins. The widespread distribution of this MFB branching pattern across the Odontoceti suggests that it arose early in the phylogeny of this group. Because the branches of the MFB consistently abut the TPC in exactly two locations, it implies that this was an important functional development. This raises two important questions: why two locations, and why these two locations? These questions will be discussed later.

### Vibrational Analysis of the Tympanoperiotic Complex (TPC) from a Bottlenose Dolphin

The vibrational analysis results indicate that the oscillations of the TPC range from simple “swinging door” motions at low frequencies (see animation linked to Figure 17) to progressively more intricate vibrational patterns as the fundamental frequency increases (see animation sequences linked to Figures 18, 19, 20, 21, 22, 23 and 24). As fundamental frequency increases, the concomitantly smaller wavelengths become smaller fractions of any particular physical dimension of the TPC. Therefore, structures large enough to support multiple wavelengths also provide interference between waves moving throughout the entire oscillating structure (the TPC in this case).

The multiformity of vibrational patterns seen in the malleus at the various resonant frequencies of the TPC (see animation sequences linked to Figures 18, 19, 20, 21, 22, 23, 24, 25, 26, 27, 28, 29, 30, 31, 32, 33 and 34) would seem to contradict the simplistic motion of the malleus proposed by Hemilä et al. [21] and Nummela et al. [28]. Hemilä et al. [21] used a lumped parameter model to investigate the action of the ossicular chain and devised a two-bone and a four-bone model. They concluded that the malleus moves along a single axis, aligned with the elongate axis of the anterior process of the malleus. Their scenarios now appear to be overly simplistic, in light of our vibrational analysis and revelations about the anatomic context of the TPC.

In their series of papers, Nummela, Hemilä, and Reuter [21], [28], [29], [52] did recognize the importance of the expansive region they termed the “tympanic plate.” They imagined it flexing along much of the region that fuses the tympanic bone to the periotic and referred to it as a “hinge.” They did not, however, identify or functionally interpret the specific loci of thin bony patches contained within the medial sulcus of the mallear ridge. By studying the TPC in isolation, without the essential soft tissue connections, Nummela et al. did not have the advantage of seeing the *anatomic context* of the TPC. Our study expands upon their work by adding the details of the soft tissue context, investigating the comparative anatomy of this system across the breadth of the Odontoceti, and applying vibrational analysis. Vibrational analysis provides an illuminating view into the displacements of various components of the system (e.g., the motion of the sigmoid process or the middle ear ossicles), which supports the functional significance of the “ear trumpet” concept. The relative motions of the various components of the TPC are clearly evident in the animated simulation sequences linked to Figures 17, 18, 19, 20, 21, 22, 23, 24, 25, 26, 27, 28, 29, 30, 31, 32, 33 and 34.

A key advancement was the study of the TPC within its anatomic context. The structural milieu of the TPC indicates a great deal about function. The TPC is acoustically isolated along its medial boundary by the peribullary sinuses and the fibrous suspension from the skull (Figure 4). The branches of the mandibular fat bodies are encapsulated in connective tissue, bounded laterally by the mandible, and function as channels for incoming sound that impinges upon the bony ear complex at specific locations. Isolating the TPC from this anatomic context removes a great number of these functional clues.

Studying the TPC in isolation may have misled Hemilä et al. [21] and Nummela et al. [28] to at least one faulty, but critical, assumption. On page 88, Hemilä et al. [21] stated, “The sound was assumed to reach the auditory bulla from an anterolateral and slightly ventral direction.” They assumed that all acoustic energy was incident upon (and orthogonal to) a single location near the thickened ventral curvature of the outer lip of the tympanic bulla, at the ventral limit of their tympanic plate (see diagram in their Figure 1, page 83). This is the same location where the large, well-known (ventral) branch of the MFB attaches to the bulla. But in *Tursiops truncatus*, it is also some 10 to 20 mm distant from the ear trumpet.

The importance of establishing clearly where the sound arrives with sufficient amplitude at the surface of the TPC lies in the following argument. Consider the task of making a seesaw move. If we push at the support, our efforts will be ineffective; on the other hand if we push at the free ends (where the seesaw undergoes large displacements), a little force will produce significant motion. Correspondingly, if sound-waves at angular frequency 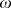 are to excite vibrations of the ear bones, the waves need to push against the surfaces of the bones, where normal modes associated with frequencies close to 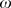 execute large displacements approximately perpendicular to the surface. The vibrational analysis animation sequences (linked to Figures 17, 18, 19, 20, 21, 22, 23, 24, 25, 26, 27, 28, 29, 30, 31, 32, 33 and 34) allowed us to inspect the relative displacements in the areas of the TPC where the branches of the MFB attach. These areas are often associated with significant displacements, areas upon which the arriving sound pressure can perform work. The work of the sound pressure on the TPC is subsequently converted into the deformation and kinetic energy of the vibrating bony ear complex. The identification of the two branches of the MFB, and of the locations where they attach to the TPC, is therefore a vital piece that helps explain the function of the middle ear. The assumption of Hemilä et al. [21] and Nummela et al. [28] is consequently determined to be an oversimplification.

The presence of the two fatty conduits may also contribute to a mechanism by which sound pressure performs work on the TPC in a slightly more subtle way. If the surfaces at which the two branches of the MFB attach move out of phase (for instance, 180° out of phase), the different lengths of the conduits may cause the sound pressure waves to also arrive with different phases. Recall the seesaw analogy: efficient simultaneous pushing at both ends would also require out of phase forces. Therefore, for some frequencies the potential for the change in phase may contribute to the mechanical functioning of the ear.

We envision the motions calculated in the vibrational analysis to represent an approximation of what might be observed in a live specimen. In fact, the vibrational analysis is based only upon the bony structure of the TPC, so there are many factors that have not yet been added to the model. For example, the properties of the ligaments that bind the ossicles together or to the oval window, or the tympanic conus/ligament that connects the malleus to the sigmoid process were not modeled. Neither have we modeled the effect of various amounts of soft tissue and/or fluid (blood engorged vascular tissue of the corpus cavernosum) that might be found in the tympanic cavity at various dive depths. However, even though these additional factors would likely change the details of the vibrational patterns, we surmise that the overall scheme would remain fairly stable, as indicated by the consistent frequency shifts found between the dry and wet mode simulations (Figure 35). What these modeling results indicate is that the vibrations of the TPC, and therefore the ossicular chain, are more complex than previously reported.

Fortunately, computer-driven FEM code is capable of solving these types of physics problems, tracking literally millions of complex structural elements, their interactions, and system characteristics (shapes, sizes, material composition, elastic properties, damping factors, etc.). As with any model of a complex system, the procedure begins with a simple representation of the problem and progressively builds-in additional complexity. The complexity is added incrementally and iteratively, with the goal of moving the model closer to approximating the actual (real) system. Fortunately, at some level, the action of the peripheral auditory system and the TPC, including the inner ear, is essentially a mechanics problem. As a consequence, it is conceivable that FEM tools could be used to calculate pressures and/or displacements within the cochlea and eventually perhaps the motions of the basilar membranes and the fluids within the cochlear ducts, leading to a reasonably complete description of the hearing apparatus whose inputs may be estimated and whose outputs are predictable.

There are other intriguing observations that can be gleaned from the calculated suite of vibrational modes (Figures 17, 18, 19, 20, 21, 22, 23, 24, 25, 26, 27, 28, 29, 30, 31, 32, 33 and 34). For example, the sigmoid process is a hollow tube that projects dorsolaterally from the tympanic bone and has a prominent elbow (Figures 10, 11, 12, 13, 14, 17, 18, 19, 20, 21, 22, 23, 24, 25, 26, 27, 28, 29, 30, 31, 32, 33 and 34). The action of the sigmoid process is particularly interesting. The vibrational analysis suggests that it often functions as a counterweight, performing different “dances” that appear reciprocal and/or synchronous with the actions of the sulcus of the mallear ridge and, through its butt joint, to the anterior process of the malleus. Of course, the precise motion patterns between various elements are nested within, and influenced by the overall pattern of vibration from all other elements of the TPC (Figures 6, 11, and 13). It is clear that the simultaneous actions of the sigmoid process and the malleus appear somewhat “connected” throughout the range of resonant frequencies that were calculated. This should be expected not only because of the bones' close proximity, but also because they are connected via the tympanic ligament or tympanic conus (Reysenbach de Haan, [53]), a remnant of the ancient tympanic membrane (Figure 11). Lancaster [54] and Fraser and Purves [55] suggested that the sigmoid process was a buttress for the malleus. Lancaster also recognized that the motion of these two components was likely partially mediated by the connection, within the tympanic cavity, between the sigmoid process and the malleus by the tympanic ligament. The sigmoid process is taxonomically diagnostic for cetaceans [42] but its morphology changes considerably across archaeocete, mysticete, and odontocete forms [54]; so any prediction about commonality of function across these groups would be mere speculation.

### Function of the middle ear

It has been approximately fifty years since Reysenbach de Haan [53] and Fraser and Purves [55] espoused that the evolutionary revamping of the middle ear was a corrective response to the selective pressures resulting from the large decrease in amplitude that occurred because of the transition between aerial and aquatic hearing environments. This supposition is perhaps not surprising because, in those early days of cetacean research, they assumed that the external auditory meatus was also functional. The problem is that their supposition ignored what happens to sounds between where they are incident upon the head and the TPC. They also lacked a proposed mechanism by which sound interacts with the ossicular chain. Previous attempts to understand this function/mechanism have been more conceptual or theoretical than experimental [23], [27], [54], [56], [57].

The two-bone sound transmission model of the ossicular chain by Nummela et al. [28] is plausible because it integrates several anatomic features into the function of the ossicles. However, in our view there are two major problems that call the model into question. Perhaps the most important problem is their proposed simplistic motion for the malleus, which they indicated is parallel to, and along the axis of the anterior process of the malleus (*processus gracilis* or gracile process), (see Fig. 5 in [28]). This is contrary to the complex family of vibrations calculated in our analysis (see animation sequences linked to Figures 25, 26, 27, 28, 29, 30, 31, 32, 33 and 34). The other major point of contention is their proposal that sound only impinges upon the TPC in one location, the outer lip of the tympanic bulla, as previously noted.

Understanding the exact mechanisms by which vibrations traverse the various components of the TPC depends on a variety of limiting factors such as, stiffness of the joint between the head of the malleus and the periotic bone, strength of the tensor tympani muscle(s)/tendon, action on the *cus breve incudis*, and the function of the tympanic ligament. The anatomy points to a complex mechanism rather than a simplistic (piston-like) motion of the malleus, as proposed by Nummela and her colleagues [29].

The posterodorsal edge of the anterior process of the malleus is fused (synostosis) by a butt joint to the thin dorsal wall of the tympanic bone ([27], p. 31; [22], p. 1423). The surrounding anatomic configuration, shown in Figures 10 and 12, with the keel between the very thinnest bony patches within the sulcus of the mallear ridge, suggests the potential for a rocking motion around the gracile process of the malleus, rather than one along its length. These displacements would be transferred through the ossicular chain. This notion is supported by the vibrational analysis, which can monitor the motion at any point during the simulations. We produced vector diagrams that represent the motion of the head of the stapes in the TPC in *Tursiops truncatus* and *Ziphius cavirostris* (Figures 36 and 37). Figures 36 and 37 display the relative magnitude and direction of the head of the stapes and demonstrate unique motions at all natural modes of vibration.

If the complex vibrational modes did not translate into unique motions of the stapes, then it could be argued that they are inconsequential. The opposite appears to be the case. These vectors make a plausible case for the fact that the complex vibrational patterns seen in the vibrational analysis could be uniquely coded in the inner ear.

The vibrational analysis produces its results from calculations based on structure. One reasonable interpretation is that the differences in the distribution of stapedial motion vectors in Figures 36 and 37 are attributable to differences in morphology between the TPC in *Ziphius cavirostris* and in *Tursiops truncatus*, which are appreciable. An inkling of these differences in structure can be gleaned from Figure 5. It is also noteworthy that nearby frequencies can result in disparate vector representations. These different vectors indicate unique motions of the footplate which in turn could generate different flow patterns within the inner ear that might be encoded to provide the animal with enhanced frequency discrimination.

### Multi-modal Stimulation of the Cochlea

One of the first observations to emerge from this analysis is a major difference between the vibrational patterns at low frequencies (e.g., Figure 17) compared to high frequencies (Figures 18, 19, 20, 21, 22, 23, 24, 25, 26, 27, 28, 29, 30, 31, 32, 33 and 34). The low frequency, long wavelength vibrations produce bulk motions of the entire TPC and little or no relative motion of the ossicles. By contrast, high frequency vibrations produce intricate vibrational patterns in the wall of the bulla that result in complex and varied motions of the individual ossicles. This is partially because higher frequencies have relatively shorter wavelengths than lower frequencies. For example, the *Tursiops* TPC is approximately 5 cm long, which is equal to the wavelength for 30 kHz in water. Consequently, frequencies below 30 kHz have a wavelength that is longer than the TPC and are likely to produces simpler interactions with it.

The vibrational analysis suggests another functional implication. The TPC appears to vibrate in two distinct modalities; one for low frequencies and one for high frequencies. The vibrational patterns at higher frequencies are complex and result in unique and relatively large amplitude motions of the stapes. Previously, these two modalities for the function of the TPC were considered mutually exclusive. But, to our knowledge they have never been considered to function as two modalities of the same TPC separated by acoustic frequency.

Conceivably, the low frequency modality results in bulk motion of the entire TPC and could lead to relative motion of the stapes caused by an inertial lag, a concept similar to the function of an otolith in a fish. The overall effect might be to reduce the response amplitude and sensitivity to low frequencies, similar to the “bone conduction” mechanism proposed by McCormick et al. [22], [24].

Hemilä et al. [21] may have conceived a similar mechanism for low frequency stimulation. Their four-bone lumped parameter model suggested a complex picture of odontocete middle ear function (see Fig. 4B, page 87 in [21]. In one scenario, they attempted to explain a curious result of McCormick et al. [22], that a cochlear response was present even after the malleus was removed. Hemilä and his colleagues assumed that the malleus is absent and consulted their model. They stated that, “It is clear that low-frequency sound will set the whole T-P complex in vibration, and the stapes will then act as an otolith vibrating in relation to the periotic bone and the cochlear capsule. Obviously, high frequency hearing and absolute sensitivity will suffer. However, low-frequency hearing may improve.” It seems clear that the surgical approach used by McCormick and his colleagues could have severely disrupted the intricate function of the TPC. As a consequence, any of their subsequent conclusions should be called into question.

We support the explanation put forth by Fleischer [27] that the surgical approach through significant amounts of vascular tissue (corpus cavernosum and the fibrous venous plexus), the trauma of breaking the malleus from its fused butt joint with the tympanic, and the potential damage to the delicate thin bones in the adjacent area, could together or separately have precipitated the odd results of McCormick et al. [22].

The dual modality function suggested by the vibrational analysis also finds support in the work of Hato et al. [58], who showed that the human stapedial footplate operates in a simple piston-like motion at low frequencies (<1.0 kHz); but moves in complex rocking motions at high frequencies. Similarly, vibrational analysis of a TPC from *Tursiops* indicated that there is little or no motion of the stapedial footplate at low frequencies (<∼20 kHz), but it exhibits more complex rocking motions at higher frequencies, often with significant displacements of the sulcus of the mallear ridge (Figures 25, 26, 27, 28, 29 30, 31, 32, 33 and 34). These complex motions are particularly visible in the accompanying animation sequences for Figures 25, 26, 27, 28, 29 30, 31, 32, 33 and 34.

In biological materials, attenuation of sound is more rapid at higher frequencies. Functionally, it follows that to compensate for the attenuated amplitudes at higher frequencies, the ossicular chain needs to be more sensitive to those attenuated frequencies, lest they be lost. There should be relatively more motion in the ossicular chain for high-frequency modes than for low-frequency modes, a characteristic that is supported by the vibrational analysis. The modes of vibrations shown here suggest that the simple ossicular motions proposed by Hemilä, Nummela, and their colleagues [21], [28] are unlikely to be applicable at the frequencies generally associated with echolocation in delphinids (>∼40 kHz).

Making the case for only bone conduction, as envisioned by McCormick and his colleagues, is difficult because it depends on the motion of the periotic with respect to the footplate of the stapes. The precise mechanism by which this is accomplished is difficult to imagine because, in most odontocetes, the periotic is to a large degree isolated from the skull by the air within the peribullary sinuses and is attached primarily by fibrous suspensory ligaments (in modern dolphins). Additionally, the stapes is held in place with an annular ligament. As a consequence, the only remaining pathway for acoustic energy to reach the periotic bone is through motion of the tympanic bone. But this is also problematic because the tympanic bone is attached to the periotic by a flexible “hinge” [21] that would severely hamper transmission of acoustic energy to the periotic bone.

The delicate attachment of the anterior process of the malleus, at the center of the thinnest patches of bone in the funnel, could be viewed as structurally similar to the terrestrial situation, where the malleus is attached to the drum-like tympanic membrane [59]. In the case of odontocetes, the thin bony membrane and the attached malleus could be expected to vibrate, even though its complexity makes it difficult to envision without vibrational analysis. Of course, there is evidence of complex, frequency dependent vibrational patterns in the ossicular chains of well known species [59], but this appears to be the first evidence of it in the highly adapted odontocete TPC, supporting the notion that the ossicular chain is functional. The result of these investigations is that sounds are literally and figuratively “knocking on the door” of the inner ear.

### How does sound excite the odontocete ear?

In his popular book, “The Porpoise Watcher” [19], Norris laid out an answer to this question and the mechanism as we now understand it: “The physicist showed that sounds hitting a thin-walled sphere produced a ‘flexural wave’ in the wall of the sphere. If the bulla is like a metal sphere, then a porpoise click echo coming down the fatty jaw channel should produce such a flexure in the bulla; it should travel around the wall of the bulla, be picked up by the thin, bony stylus of the first ear ossicle, and be transmitted thence to the inner ear and brain” ([19], p. 213). A decade earlier [7], Norris had already recognized the primacy of fatty tissue for sound transmission and reception in the odontocete head. Our analysis has not produced evidence for a traveling wave propagating around the wall, as Norris suggested, but otherwise, Norris' notion of complex flexing in the wall of the TPC is supported by our work.

The issue of impedance matching may initially or intuitively suggest that acoustic energy will not enter the TPC by way of a bony interface with fatty channels, and eventually find a bony pathway to the inner ear. But, it is essential to keep in mind that the cone-shaped dorsal branch of each mandibular fat body attaches to the TPC where there are multiple thinned bony elements (Figure 13). The question immediately arises, what mechanism could transduce acoustic energy from the least dense tissue in the body (fat) to the densest tissue known (pachyostotic bone)?

Here we posit a mechanism that seems closely aligned with Norris' proposal [19]. Consider a couple of important factors. First, the extreme impedance mismatch at the interface between the fatty attachments of the MFB and the bony elements of the TPC is essential, because it causes the greatest possible force to be exerted upon the bone from incoming sound pressure reflections. Second, the thinness of the bony regions presumably increases flexibility locally and increases the likelihood that any bending will occur there.

We envision that the thinned regions of bone in the TPC represent zones of differential flexibility, whose actions collectively result in intricate vibrational patterns (Figures 17, 18, 19, 20, 21, 22, 23, 24, 25, 26, 27, 28, 29, 30, 31, 32, 33 and 34 and the accompanying animation sequences). Accordingly, the various patches of thinned bone within the TPC (Figure 13), are integral components of an elaborate transduction mechanism. We assume it is not a coincidence that the two thinnest bony patches are adjacent to the elongate joint between the anterior process of the malleus and tympanic bone (Figures 10, 11 and 13).

Our proposal is that acoustic signals enter over the surface of the head, are variously filtered or amplified by anatomic components, while propagating to and through the MFB, and eventually exert sound pressure across a mosaic of bony patches of varying thinness. The sound pressures are summed across the mosaic of bony elements and are transduced into mechanical displacements that result in complex vibrations of the entire TPC, including the ossicular chain (Figures 25, 26, 27, 28, 29 30, 31, 32, 33 and 34). If this proposal for the transduction mechanism is correct, it follows that the patterns of sound pressure on the actuated bony surfaces of the TPC are so complex that the intricate vibrational interactions can only be seen with the aid of computers.

### Conclusions

The current paper provides answers to a group of pivotal questions related to the structure/function complex of the odontocete TPC: 1) what are the probable sites and mechanisms for acoustic stimulation of the TPC? 2) Do these results suggest whether or not the ossicular chain is functional? 3) If the middle ear is functional, how might sound pressure be transmitted through the TPC to the cochlea as vibrational motions or displacements?

The vibrational analysis represents a leap forward in experimentation with this complex system. It shows that the TPC, with bones of varying thicknesses, joints, and soft tissues, is capable of fundamental vibrational states that are more complex than previously reported. The absence of knowledge about the acoustic input to the TPC is another major deficit in the previous attempts to understand the osseous system of the TPC, using lumped parameter models and other conceptual processes (e.g., Hemilä et al. [21]). Some of the FEM simulations with *Ziphius cavirostris* [60] suggested that there is a primary pathway for sound that reaches the TPC via the gular anatomy.

This pathway is only possible because of the absence of the medial wall of the posterior mandibles, the “open door” that acoustically exposes the mandibular fat body leading to the TPC. Since all extant odontocetes are similarly constructed, they may all use this same general pathway. By implication, a similar acoustic pathway may have been functional in the ancient whales because the fossil record shows that archaeocetes also exhibit an excavated posterior mandible, perhaps the rudiments of the “open door.”

The FEM techniques employed here promise a window into acoustic mechanisms and a new vista for virtual experimentation. Propagation models are currently being conducted with other odontocete species to test for the presence of an internal acoustic pinna, which might supplement the amplification function. Amplification, normally a primary function of the middle ear, is difficult to determine in odontocetes, particularly because the apparatus is so inaccessible and the acoustic environment within the body of an aquatic animal is prohibitively complex to sort out experimentally.

Combining CT imaging, tissue property measurements, and FEM provided a foundation for constructing a modeling environment (the vibroacoustic toolkit), producing a variety of computer simulations that either verified prior results and speculation, or produced novel results and potential discoveries. This may be particularly true with respect to the vibratory function of the TPC and the middle ear.

Our project is not the first attempt to understand the function of the TPC but it is the first to employ a technique with the capacity to unravel the intricately intertwined family of vibrational patterns that result from the structural complexity inherent in the odontocete TPC. It provided the first opportunity to simultaneously visualize the relative motions of various anatomic components (i.e., the individual ossicles, the sigmoid process, the floor of the sulcus of the mallear ridge, etc.).

If our new propagation model of the entire head of a bottlenose dolphin shows acoustic pressure directed to the locations where the fatty branches attach to the TPC, then significant displacements of the underlying bony regions should be expected and the ear trumpet function will be confirmed.

## Supporting Information

Figure S17 Animated GIF for Figure 17. At this first natural mode of vibration (8.1 kHz), the motion is large, low-frequency swinging movements.
(1.09 MB GIF)

Figure S18 Animated GIF for Figure 18. This animation shows the 33rd natural mode of vibration (65.5 kHz). Note that some of the largest displacements occur in the medial sulcus of the mallear ridge. In addition, the adjacent sigmoid process is similarly active.
(1.11 MB GIF)

Figure S19 Animated GIF for Figure 19. This animation shows the 40th natural mode of vibration (76.8 kHz). As the frequency rises, the wavelength gets smaller, allowing a greater number of complete cycles (peaks and valleys) to be supported across the TPC.
(1.18 MB GIF)

Figure S20 Animated GIF for Figure 20. This animation shows the 53rd natural mode of vibration (92.5 kHz). It illustrates the nature of the “counterbalancing” or “compensating” motions of sigmoid process and the medial sulcus of the mallear ridge.
(1.18 MB GIF)

Figure S21 Animated GIF for Figure 21. This animation shows the 56th natural mode of vibration (96.7 kHz). The vibrational patterns continue to get more complex as frequency increases.
(1.19 MB GIF)

Figure S22 Animated GIF for Figure 22. This animation shows the 63rd natural mode of vibration (104.8 kHz). It is interesting to observe that the higher frequencies are associated with relatively larger amplitudes of motion across the stapes (see Figure 36). One may conjecture that a mechanism like this may have evolved to compensate for the attenuation of high frequencies in biological tissues.
(1.27 MB GIF)

Figure S23 Animated GIF for Figure 23. This animation shows the 65th natural mode of vibration (107.5 kHz).
(1.18 MB GIF)

Figure S24 Animated GIF for Figure 24. This animation shows the 117th natural mode of vibration (157.8 kHz) for this Tursiops truncatus TPC. This frequency is at the upper end of the useable acoustic range for this species, according to the literature. It is also nearly the highest mode we calculated for this TPC.
(1.21 MB GIF)

Figure S25 Animated GIF for Figure 25. In this view, the TPC has been turned upside down and the medial side removed so that the middle ear ossicles are visible. This animation shows the relatively small motions of ossicles for the 11th natural mode of vibration (32.1 kHz).
(0.73 MB GIF)

Figure S26 Animated GIF for Figure 26. In this view, the TPC has been turned upside down and the medial side removed so that the middle ear ossicles are visible. This animation shows the ossicular motion for the 34th natural mode of vibration (67.1 kHz), where they move in relative unison.
(0.72 MB GIF)

Figure S27 Animated GIF for Figure 27. In this view, the TPC has been turned upside down and the medial side removed so that the middle ear ossicles are visible. This animation shows that the ossicles move in unison for the 40th natural mode of vibration (76.8 kHz), but in a different direction than in previous modes.
(0.80 MB GIF)

Figure S28 Animated GIF for Figure 28. In this view, the TPC has been turned upside down and the medial side removed so that the middle ear ossicles are visible for the 49th natural mode of vibration (87.7 kHz). This animation example shows that the ossicles begin to move with slight twisting motions with respect to one another.
(0.78 MB GIF)

Figure S29 Animated GIF for Figure 29. In this view, the TPC has been turned upside down and the medial side removed so that the middle ear ossicles are visible for the 56th natural mode of vibration (96.7 kHz). This example shows that the ossicles move with more exaggerated twisting motions with respect to one another.
(0.79 MB GIF)

Figure S30 Animated GIF for Figure 30. In this view, the TPC has been turned upside down and the medial side removed so that the middle ear ossicles are visible for the 64th natural mode of vibration (105.7 kHz). This example shows that the ossicles move with multiple extreme twisting motions with respect to one another.
(0.81 MB GIF)

Figure S31 Animated GIF for Figure 31. In this view, the TPC has been turned upside down and the medial side removed so that the middle ear ossicles are visible for the 68th natural mode of vibration (109.3 kHz). This example shows that the ossicles move with different twisting trajectories with respect to previous examples.
(0.72 MB GIF)

Figure S32 Animated GIF for Figure 32. In this view, the TPC has been turned upside down and the medial side removed so that the middle ear ossicles are visible for the 79th natural mode of vibration (122.3 kHz). In this example the malleus and incus are once again moving in unison with one another.
(0.77 MB GIF)

Figure S33 Animated GIF for Figure 33. In this view, the TPC has been turned upside down and the medial side removed so that the middle ear ossicles are visible for the 102nd natural mode of vibration (143.8 kHz). In this example the malleus twists in an entirely new rotational axis with respect to the incus.
(0.79 MB GIF)

Figure S34 Animated GIF for Figure 34. In this view, the TPC has been turned upside down and the medial side removed so that the middle ear ossicles are visible for the 105th natural mode of vibration (146.4 kHz). This example shows the most extreme twisting displacements of the ossicles.
(0.80 MB GIF)

Appendix S1 Details on the formulation of the model.
(0.06 MB DOC)
