# Supplementary figures and images for: A New Acoustic Portal into the Odontocete Ear and Vibrational Analysis of the Tympanoperiotic Complex
Source: PLoS One. 2010 Aug 4;5(8):e11927. doi: 10.1371/journal.pone.0011927 (PMC2915923; doi:10.1371/journal.pone.0011927)

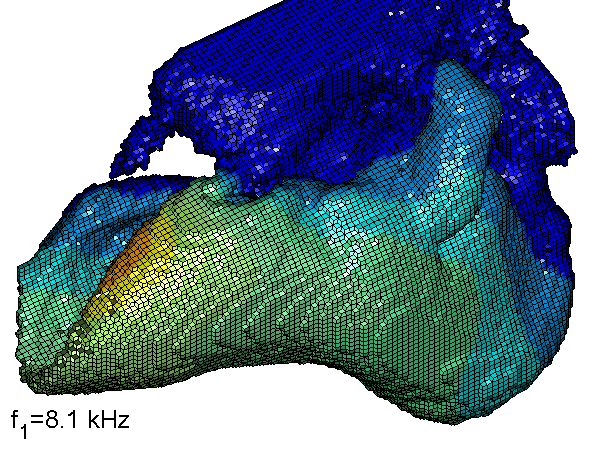

Supplement: Figure S17 — Animated GIF for Figure 17. At this first natural mode of vibration (8.1 kHz), the motion is large, low-frequency swinging movements. (1.09 MB GIF) [file pone.0011927.s001.gif]

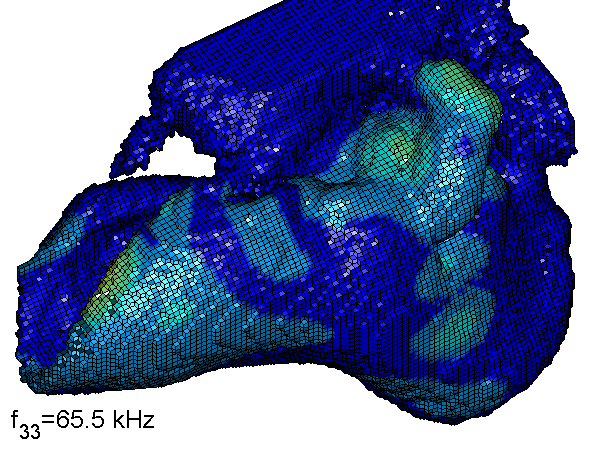

Supplement: Figure S18 — Animated GIF for Figure 18. This animation shows the 33rd natural mode of vibration (65.5 kHz). Note that some of the largest displacements occur in the medial sulcus of the mallear ridge. In addition, the adjacent sigmoid process is similarly active. (1.11 MB GIF) [file pone.0011927.s002.gif]

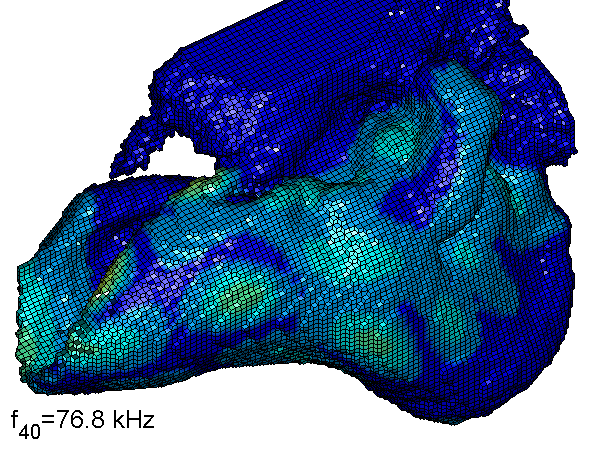

Supplement: Figure S19 — Animated GIF for Figure 19. This animation shows the 40th natural mode of vibration (76.8 kHz). As the frequency rises, the wavelength gets smaller, allowing a greater number of complete cycles (peaks and valleys) to be supported across the TPC. (1.18 MB GIF) [file pone.0011927.s003.gif]

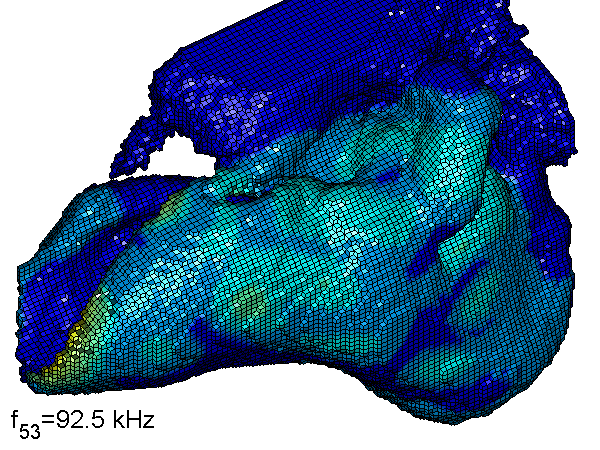

Supplement: Figure S20 — Animated GIF for Figure 20. This animation shows the 53rd natural mode of vibration (92.5 kHz). It illustrates the nature of the “counterbalancing” or “compensating” motions of sigmoid process and the medial sulcus of the mallear ridge. (1.18 MB GIF) [file pone.0011927.s004.gif]

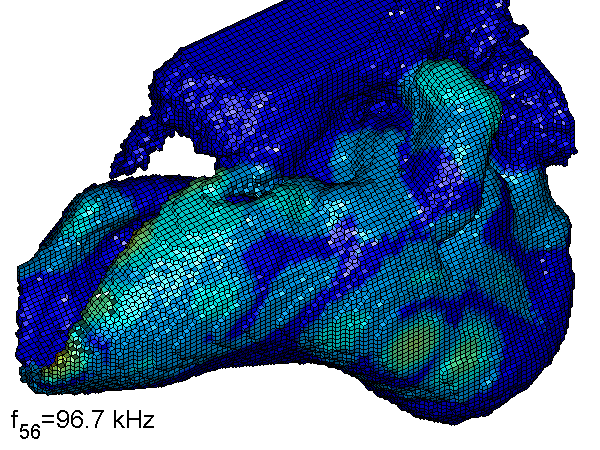

Supplement: Figure S21 — Animated GIF for Figure 21. This animation shows the 56th natural mode of vibration (96.7 kHz). The vibrational patterns continue to get more complex as frequency increases. (1.19 MB GIF) [file pone.0011927.s005.gif]

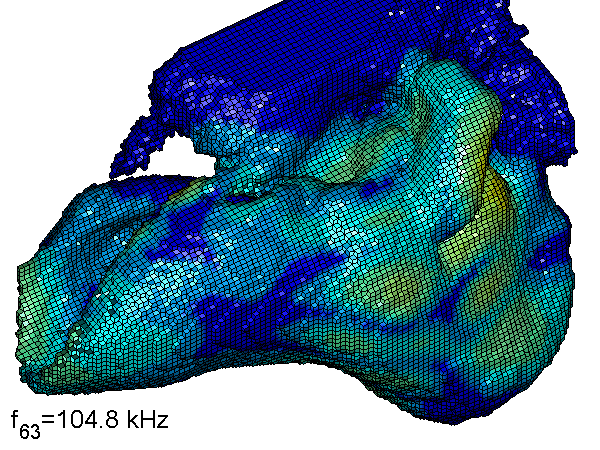

Supplement: Figure S22 — Animated GIF for Figure 22. This animation shows the 63rd natural mode of vibration (104.8 kHz). It is interesting to observe that the higher frequencies are associated with relatively larger amplitudes of motion across the stapes (see Figure 36). One may conjecture that a mechanism like this may have evolved to compensate for the attenuation of high frequencies in biological tissues. (1.27 MB GIF) [file pone.0011927.s006.gif]

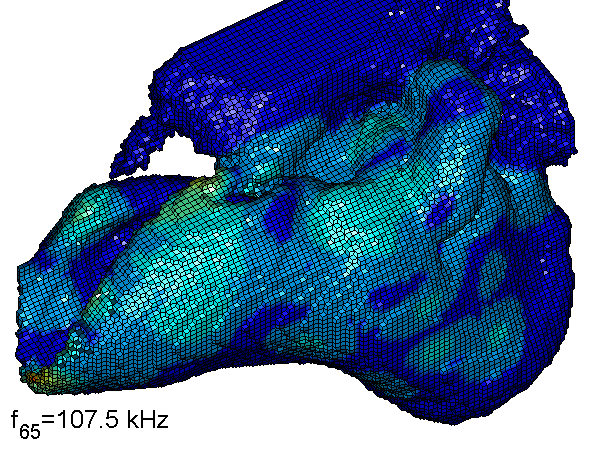

Supplement: Figure S23 — Animated GIF for Figure 23. This animation shows the 65th natural mode of vibration (107.5 kHz). (1.18 MB GIF) [file pone.0011927.s007.gif]

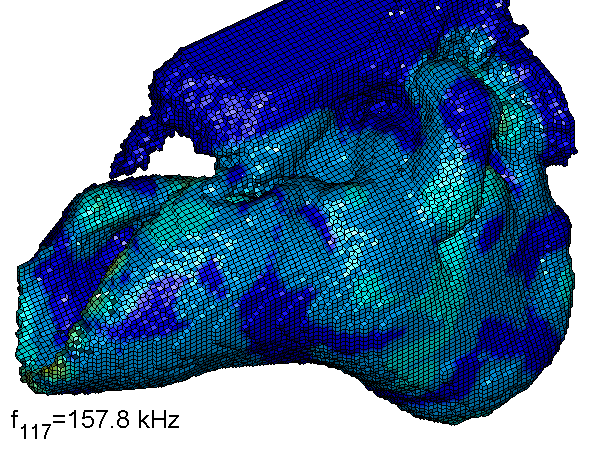

Supplement: Figure S24 — Animated GIF for Figure 24. This animation shows the 117th natural mode of vibration (157.8 kHz) for this Tursiops truncatus TPC. This frequency is at the upper end of the useable acoustic range for this species, according to the literature. It is also nearly the highest mode we calculated for this TPC. (1.21 MB GIF) [file pone.0011927.s008.gif]

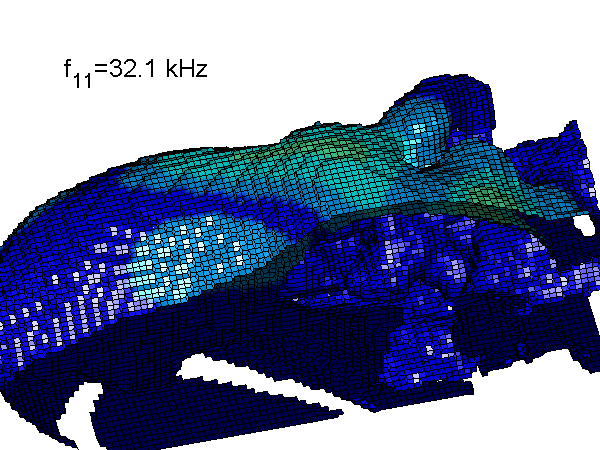

Supplement: Figure S25 — Animated GIF for Figure 25. In this view, the TPC has been turned upside down and the medial side removed so that the middle ear ossicles are visible. This animation shows the relatively small motions of ossicles for the 11th natural mode of vibration (32.1 kHz). (0.73 MB GIF) [file pone.0011927.s009.gif]

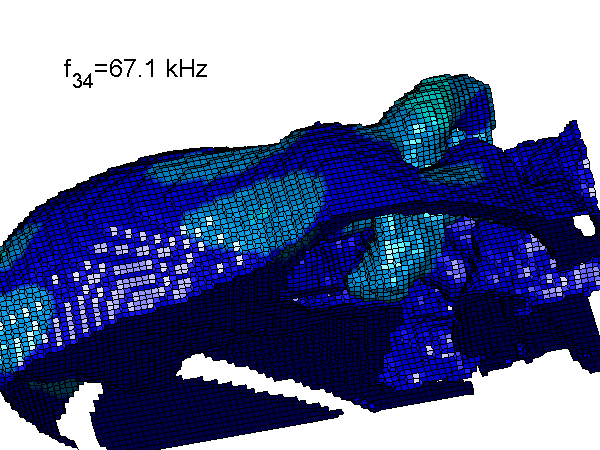

Supplement: Figure S26 — Animated GIF for Figure 26. In this view, the TPC has been turned upside down and the medial side removed so that the middle ear ossicles are visible. This animation shows the ossicular motion for the 34th natural mode of vibration (67.1 kHz), where they move in relative unison. (0.72 MB GIF) [file pone.0011927.s010.gif]

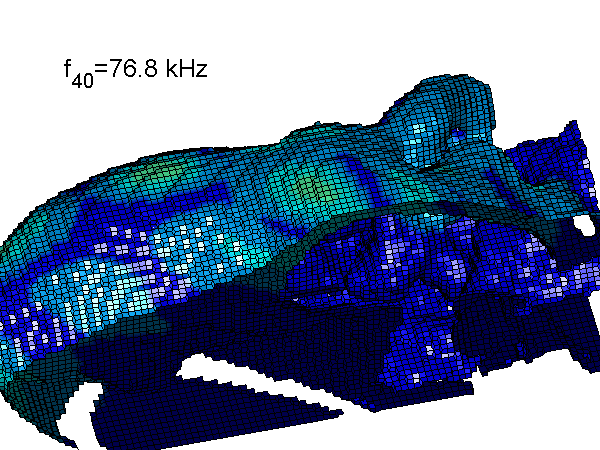

Supplement: Figure S27 — Animated GIF for Figure 27. In this view, the TPC has been turned upside down and the medial side removed so that the middle ear ossicles are visible. This animation shows that the ossicles move in unison for the 40th natural mode of vibration (76.8 kHz), but in a different direction than in previous modes. (0.80 MB GIF) [file pone.0011927.s011.gif]

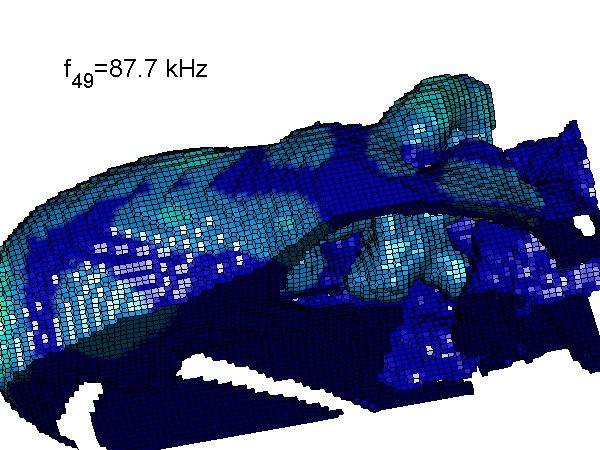

Supplement: Figure S28 — Animated GIF for Figure 28. In this view, the TPC has been turned upside down and the medial side removed so that the middle ear ossicles are visible for the 49th natural mode of vibration (87.7 kHz). This animation example shows that the ossicles begin to move with slight twisting motions with respect to one another. (0.78 MB GIF) [file pone.0011927.s012.gif]

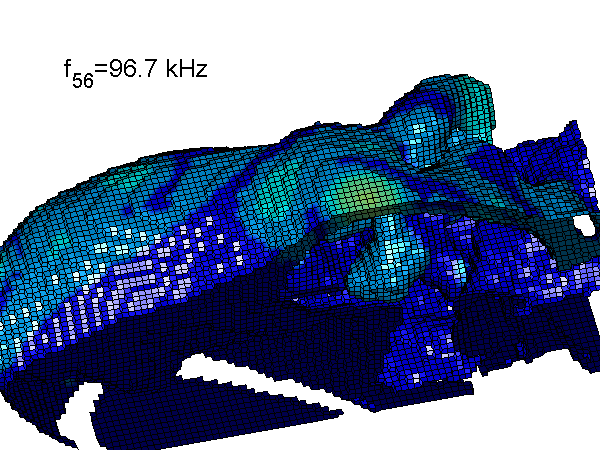

Supplement: Figure S29 — Animated GIF for Figure 29. In this view, the TPC has been turned upside down and the medial side removed so that the middle ear ossicles are visible for the 56th natural mode of vibration (96.7 kHz). This example shows that the ossicles move with more exaggerated twisting motions with respect to one another. (0.79 MB GIF) [file pone.0011927.s013.gif]

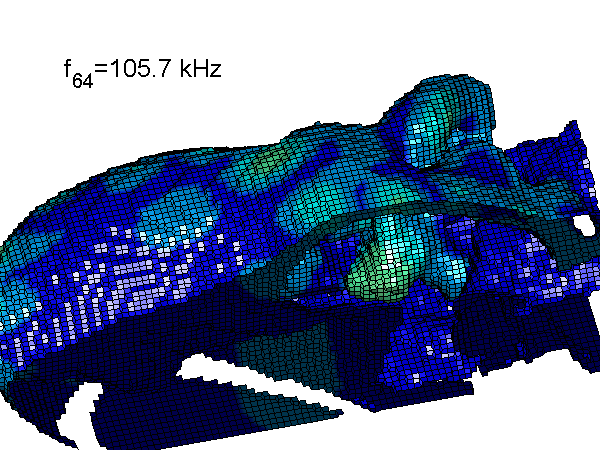

Supplement: Figure S30 — Animated GIF for Figure 30. In this view, the TPC has been turned upside down and the medial side removed so that the middle ear ossicles are visible for the 64th natural mode of vibration (105.7 kHz). This example shows that the ossicles move with multiple extreme twisting motions with respect to one another. (0.81 MB GIF) [file pone.0011927.s014.gif]

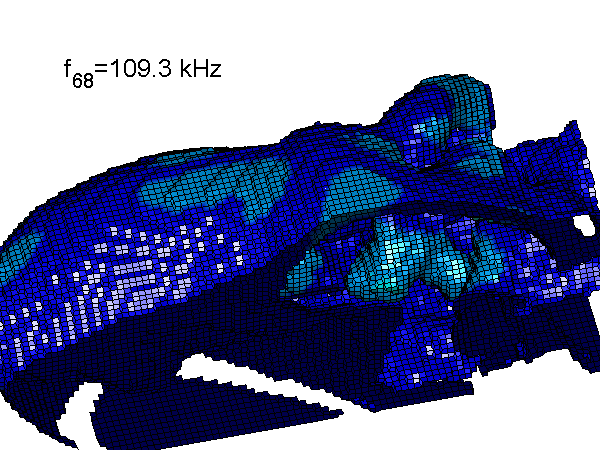

Supplement: Figure S31 — Animated GIF for Figure 31. In this view, the TPC has been turned upside down and the medial side removed so that the middle ear ossicles are visible for the 68th natural mode of vibration (109.3 kHz). This example shows that the ossicles move with different twisting trajectories with respect to previous examples. (0.72 MB GIF) [file pone.0011927.s015.gif]

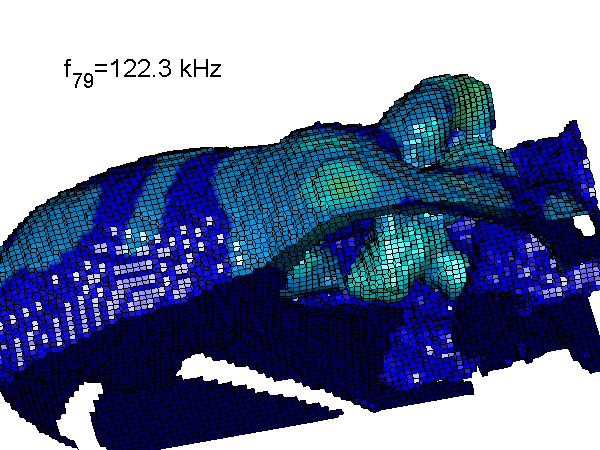

Supplement: Figure S32 — Animated GIF for Figure 32. In this view, the TPC has been turned upside down and the medial side removed so that the middle ear ossicles are visible for the 79th natural mode of vibration (122.3 kHz). In this example the malleus and incus are once again moving in unison with one another. (0.77 MB GIF) [file pone.0011927.s016.gif]

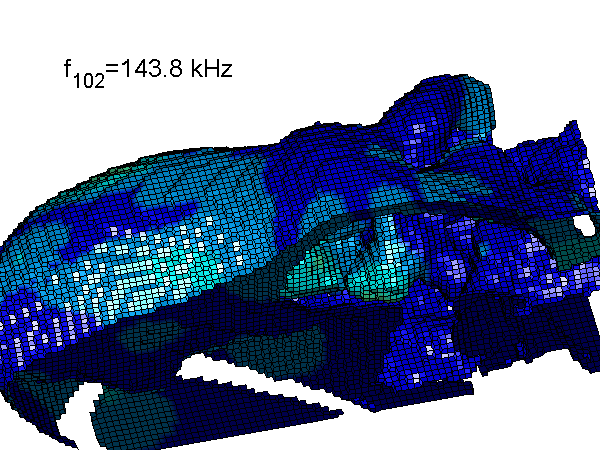

Supplement: Figure S33 — Animated GIF for Figure 33. In this view, the TPC has been turned upside down and the medial side removed so that the middle ear ossicles are visible for the 102nd natural mode of vibration (143.8 kHz). In this example the malleus twists in an entirely new rotational axis with respect to the incus. (0.79 MB GIF) [file pone.0011927.s017.gif]

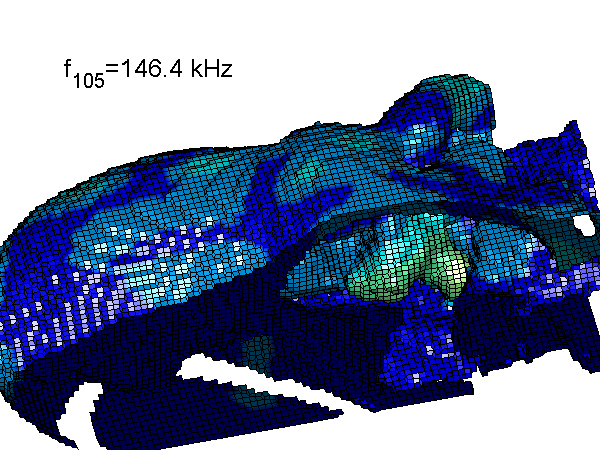

Supplement: Figure S34 — Animated GIF for Figure 34. In this view, the TPC has been turned upside down and the medial side removed so that the middle ear ossicles are visible for the 105th natural mode of vibration (146.4 kHz). This example shows the most extreme twisting displacements of the ossicles. (0.80 MB GIF) [file pone.0011927.s018.gif]
