# Supplementary material for: A New Acoustic Portal into the Odontocete Ear and Vibrational Analysis of the Tympanoperiotic Complex
Source: PLoS One. 2010 Aug 4;5(8):e11927. doi: 10.1371/journal.pone.0011927 (PMC2915923; doi:10.1371/journal.pone.0011927)
Supplement: Appendix S1 — Details on the formulation of the model. (0.06 MB DOC) [file pone.0011927.s019.doc]

**Appendix:**

The assumption that the surrounding tissue can be modeled as an incompressible fluid needs to be carefully assessed. The reactive force due to the added mass may be considered to be adequately represented by incompressible-fluid models in situations in which the wave number in the vibrating solid is much larger than the wave number in the surrounding fluid. Approximating the vibrating thin parts of the tympanic bone as thin plates (after [61]) the bending wave number, $k_{b}$ was obtained as:

$$k_{b}=\sqrt{\omega}\left(\rho h/D\right)^{1/4}$$

where $\rho$ and $h$ are the mass density and the thickness of the plate, $\omega$ is the circular frequency, and $D$ is the bending stiffness

$D=\frac{Eh^{3}}{12(1-\nu^{2})}$.

Thus we may estimate the ratio of the fluid wave number $k_{w}=\omega/c_{w}$ (where $c_{w}$is the speed of sound in the fluid, here taken to be representative of the acoustic fats) and the solid wave number $k_{b}$can be estimated. The assumption that the added mass may be accurately modeled by an incompressible fluid becomes inaccurate for natural frequencies approaching 100 kHz because $k_{w}$ grows faster than $k_{b}$. On the other hand $k_{b}$ estimate above is for a flat plate, whereas the tympanic bone is almost always a doubly curved shell, with correspondingly larger wave numbers. The impact of such errors may however be limited since the influence of the surrounding fluid on the *in vacuo* modes decreases as vibration frequencies increase [61]. The mode shapes are largely unaffected by the presence of fluid, and the corresponding natural frequencies decrease as

$$\omega_{j,wet}\approx\omega_{j,dry}\left(1+\frac{\rho_{w}}{\rho hk_{b}}\right)^{-1/2}$$

Further we may refer to the empirical formulas proposed by Blevins [62]. The potential flow theory (that is the incompressible inviscid fluid model) may be expected to furnish results in good agreement with experiments provided the following three conditions are met:

- The displacement amplitude, $X$, must be much smaller than the characteristic dimension of the vibrating body, $L=40 \text{mm}$. This condition is met since we expect the oscillatory amplitude in response to acoustic excitation $X$ is on the order of micrometers, and furthermore our vibrational model is linear.
- The Reynolds-number-like parameter should be large
  $\frac{\rho_{w}\omega L^{2}}{\mu_{w}}\geq10^{4}$.
  Here $\mu_{w}$ is the dynamic viscosity. We take as an approximation frequency of 20 kHz, the viscosity of generic soft tissue at this frequency , $\mu_{w}=20 \text{Pa}\cdot\text{s}^{\text{-1}}$, and the characteristic dimension $L=40 \text{mm}$. Then to obtain $\frac{\rho_{w}\omega L^{2}}{\mu_{w}}\approx9047$.
- The oscillatory Mach number must be small
$\frac{X\omega}{c_{w}}<<1.$
  This condition is met for the lowest natural frequency in the system of approximately 8 kHz, and the displacement amplitude on the order of 1 µm
$\frac{X\omega}{c_{w}}=3.6\times10^{-5}<<1.$

In conclusion, while the incompressible-fluid model may be crude at the high-frequency range, the errors are not likely to change the qualitative picture of the dynamic mechanical system of the ear bone. In the future, a more sophisticated vibroacoustic model may be used to take into account the compressibility of the surrounding tissue and possibly also other effects that have been neglected, such as shear elasticity of the tissue and attenuation both in the tissue and in the bone.
